# Targeting the tumor stroma for cancer therapy

**DOI:** 10.1186/s12943-022-01670-1

**Published:** 2022-11-02

**Authors:** Maosen Xu, Tao Zhang, Ruolan Xia, Yuquan Wei, Xiawei Wei

**Affiliations:** Laboratory of Aging Research and Cancer Drug Target, State Key Laboratory of Biotherapy, West China Hospital, National Clinical Research Center for Geriatrics, Sichuan University, No. 17, Block 3, Southern Renmin Road, 610041 Chengdu, Sichuan PR China

**Keywords:** Tumor stroma, Extracellular matrix, cancer-associated fibroblasts, Mesenchymal stromal cells, Pericytes, cancer therapy, Targeted therapy, Clinical trial

## Abstract

Tumors are comprised of both cancer cells and surrounding stromal components. As an essential part of the tumor microenvironment, the tumor stroma is highly dynamic, heterogeneous and commonly tumor-type specific, and it mainly includes noncellular compositions such as the extracellular matrix and the unique cancer-associated vascular system as well as a wide variety of cellular components including activated cancer-associated fibroblasts, mesenchymal stromal cells, pericytes. All these elements operate with each other in a coordinated fashion and collectively promote cancer initiation, progression, metastasis and therapeutic resistance. Over the past few decades, numerous studies have been conducted to study the interaction and crosstalk between stromal components and neoplastic cells. Meanwhile, we have also witnessed an exponential increase in the investigation and recognition of the critical roles of tumor stroma in solid tumors. A series of clinical trials targeting the tumor stroma have been launched continually. In this review, we introduce and discuss current advances in the understanding of various stromal elements and their roles in cancers. We also elaborate on potential novel approaches for tumor-stroma-based therapeutic targeting, with the aim to promote the leap from bench to bedside.

## Introduction

Although tremendous progress has been achieved, cancer remains a multifactorial disease with limited therapeutic strategies and one of the leading causes of premature death. In 2020, there were an estimated 19.3 million new cancer cases and approximately 10.0 million deaths caused by cancer worldwide [1], which indicated that malignant tumors seriously threaten public health. Therefore, it is necessary to comprehensively investigate the sophisticated pathogenesis of malignancies and develop effective approaches for cancer treatment.

Dating back to the 1880s, Stephen Paget proposed the “seed and soil” hypothesis and revealed that certain tumor cells displayed preferential affinity to invade specific organs, highlighting the critical role of the microenvironment in regulating metastasis growth [2, 3]. Nowadays, it is widely accepted that the tumor microenvironment (TME) constitutes the immediate niche surrounding tumor tissues and is implicated in tumorigenesis [4, 5]. As an essential element of the TME, the tumor stroma affects tumor biology and contributes to cancer initiation, progression, metastasis, and therapeutic resistance [6].

The tumor stroma is highly dynamic, heterogeneous and commonly tumor-type specific. It is mainly composed of noncellular compositions such as the extracellular matrix (ECM) and the unique cancer-associated vascular system as well as a diverse cellular components including, but not limited to, activated cancer-associated fibroblasts (CAFs), mesenchymal stromal cells (MSCs), pericytes [7–12]. These abundant stromal components form a dynamic milieu to support cancer progression and can potentially be regarded as biomarkers in cancer [13]. Importantly, the low tumor-stroma ratio (TSR) is remarkably correlated with poorer survival outcomes, and the TSR can be a valuable predictor for evaluating the prognosis and treatment outcome of cancer patients [14–18]. Except for tumor-promoting actions, stromal components can also restrain tumor growth, especially in pancreatic ductal adenocarcinoma, because the complete ablation of stroma resulted in a more invasive tumor phenotype and reduced overall survival [19–22]. In the early stages of tumorigenesis or metastatic dissemination, the stroma can be considered tumor suppressive [6]. However, the tumor stroma is constantly changing rather than a static entity, and our researches mainly focus on the roles and mechanisms by which stromal elements accelerate cancer initiation and progression, aiming at providing theoretical rationales and preclinical evidence for tumor-stroma-targeted therapy [23–25].

Over the past few decades, we have witnessed an exponential increase in the investigation and recognition of the critical role of stroma in solid tumors. Coupled with the significant progress of new insights to explore intrastromal communication, we are beginning to see the deployment of stroma-targeted cancer therapy. A series of clinical trials targeting the tumor stroma have been launched continually. In this review, we detailed introduce current advances in the understanding of various stromal elements and their roles in cancer. Furthermore, we summarize recent knowledge regarding the interplay between those various stromal compartment and elaborate on potential approaches for tumor-stroma-based therapeutic targeting.

## Components of the tumor stroma

Tumor tissue is a heterogeneous mixture of both cancer cells and various stromal components. In solid tumors, stromal elements interact with neoplastic cells to influence tumor behavior. Tumor cells can alter their surrounding stroma, forming a permissive microenvironment to support their growth. Interestingly, tumor cells can also transdifferentiate into stromal-like cells through different signal transduction pathways to enhance tumor angiogenesis and facilitate cancer development [26–28].

**Fig. 1:**
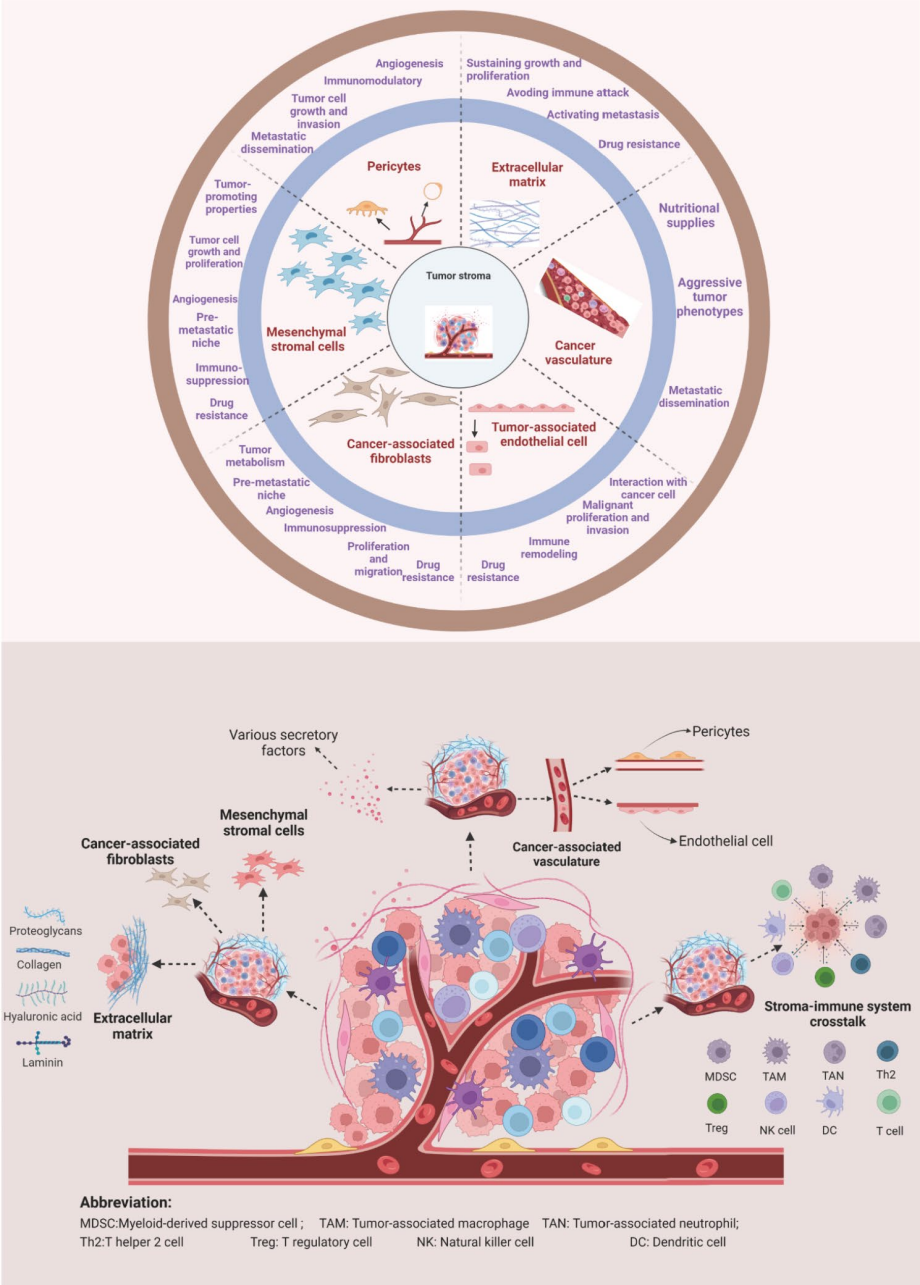
Major components of the tumor stroma and their tumor-promoting functions

The tumor stroma participates in tumorigenesis, cancer progression, and therapy resistance, and it also profoundly affects many hallmarks of cancer [29–31]. Stromal elements contain the ECM, vasculature, and various cellular components such as activated CAFs, MSCs, pericytes, and osteoblasts. These components affect anti-tumor immune and determine neoplastic progression (Fig. 1). For example, osteoblasts are responsible for attracting cancer cells to bone marrow and driving malignant cells’ bone metastasis [32]. Adipocytes, as a population of active facilitators, affect cancer metabolism and are involved in tumor establishment, progression, and therapeutic resistance [33–36]. In recent years, oncologists have investigated the functions of osteoblasts and adipocytes in cancer, but their detailed description is beyond this Review’s scope. Herein, we mainly focus on the functions of ECM, stromal vasculature, tumor-associated endothelial cells, CAFs, MSCs, and pericytes.

### Extracellular matrix (ECM)

The extracellular matrix has a pivotal role in modulating and maintaining tissue development and homeostasis, but the dysregulation and mechanical features of ECM can determine cancer aggressiveness and impact the sensitivity to drug therapy [37–39]. The altered and stiffened ECM affects virtually every facet of cancer hallmarks including avoiding immune destruction, tumor-promoting inflammation, activating invasion and metastasis, and inducing angiogenesis [29, 40–42]. Therefore, the ECM not only influences the tumor behavior and histopathology but also be regarded as an integral and remarkable feature of cancer [43].

The ECM is an intricate and dynamic structure that is constantly remodeled by the synthesis and degradation of numerous ECM proteins [44, 45]. In general, the complex ECM network consists of fibrillar or non-fibrillar collagens, proteoglycans, glycoproteins, laminins, fibronectins and other macromolecules. Among them, collagens are the most abundant components of ECM [46]. Commonly, the deregulation of ECM homeostasis leads to cancer evolvement through two distinct mechanisms. On the one hand, ample molecules mainly derived from CAFs induce the pro-fibrotic response and result in excessive deposition of ECM, thereby protecting tumor cells from immune destruction and mediating therapeutic resistance. On the other hand, continuous ECM breakdown contributes to reducing the cancerous cell-ECM adhesion, promoting tumor cells’ invasive and migratory abilities, and inducing malignant cells intravasation via the regulation of invadopodia formation [47–51].

It is now accepted that excessive deposition of collagen and crosslinking of fibrillar collagens and elastin result in the dense ECM and increase the stroma stiffness, which has profound impacts on cancer progression [52]. Increased ECM deposition represents a crucial physical barrier that inhibits antitumor immunity [53]. Apart the formation of a natural barrier, the stiff ECM can also increase the expression of PD-L1 in lung cancer cells in a actin-dependent manner, thereby protecting tumor cells from the host immune attack [54]. The ECM together with tumor cell’s architecture also constitute a physical barrier for drug delivery [55]. In PDAC, stiffened ECM can reduce vascular density and induce epithelial-mesenchymal-transition, which results in the embeddedness of vessels into the matrix and subsequently creates a tough barrier to prevent drug perfusion [56, 57]. The stiff matrix can compress the micro blood vessels, and thus impedes the successful access of anti-tumor drugs into core tumor tissues through the vasculature [58, 59]. Tumor cells surrounded by the stroma can adhere to various ECM proteins, which decreases the chemotherapy efficacy, known as cell adhesion-mediated drug resistance [60]. Intriguingly, stiffened ECM can mechanoactivate glycolysis and glutamine metabolism to coordinate the flux of nonessential amino acid in the tumor tissue, which modulates tumor metabolism and potentially provides energies for tumor growth and aggressiveness [61]. Moreover, abundant ECM deposition potentiates the adhesion of metastatic malignant cells to the tumor endothelium, thus promoting cancer intravasation and subsequent metastasis [62].

Tumor cells often exhibit higher mobility in the remodeled ECM. Simultaneously, the remodeled ECM facilitates cancer cell-directed migration toward the vasculature, favoring the metastatic dissemination of these cells [63, 64]. When integrin binds to its ECM ligand, the FAK/Src complex is assembled at the cytoplasmic tail of integrin, which promotes the activation of downstream signals PI3K/AKT and RAS/MEK/ERK circuits to maintain cell survival and migration [65, 66]. Ras Suppressor-1 (RSU-1) is a cell-ECM protein and is obviously upregulated in breast cancer cells embedded in stiffer 3D collagen I gels. RSU-1 silencing resulted in the inhibition of MMP-13 and urokinase plasminogen activator, thereby reducing cancer cell invasion and migration [67]. Furthermore, some matrix metalloproteinases (MMPs) can degrade the ECM network, which mediates the pro-invasive phenotype of cancer cell and augments the cell mobility throughout the ECM [68, 69].

MMPs belong to ECM proteins that are involved in nearly all important steps during carcinogenesis and progression. More than 20 MMPs have been identified so for and most of them exist in the human proteome [70]. The activity of these enzymes is low under normal circumstances, but in the setting of tumor development their activity can be increased. Among all these MMP family numbers, MMP9 and MMP2 are perhaps the best-studied type and they can degrade the IV collagen to regulate ECM remodeling [71, 72]. MMP9 can also accelerate angiogenesis, tumor invasion and metastasis. Given its important role in tumorigenesis, MMP9 is currently considered as a biomarker and a legitimate therapeutic target for many cancer types [73]. Homoplastically, MMP2 induces tumor neovascularization through the activation of pro-angiogenic factors such as vascular endothelial growth factor (VEGF) and TGF-β, and it also promotes the proteolytic degradation of extracellular proteins to drive tumor metastasis [74, 75]. Apart from MMP9 and MMP2, other MMPs that are present in ECM also have tight association with oncogenic advancement. For example, MMP1, MMP3 and MMP10 have been found to promote cancer cell initial invasion and distant dissemination. MMP7 and MMP13 contribute to tumor cell growth and proliferation. Moreover, some MMPs can prevent the apoptosis of early cancer cell, such as MMP7, MMP10 and MMP11 [74]. An alternative key function of MMPs in cancer metastasis is to regulate the formation of invadopodia that is implicated in breaching basement membrane ao as to allow the extravasation and movement of tumor cell through tissues [76–78]. The targeted delivery and exocytosis of MMP2, MMP9 and MMP14 is required for invadopodia maturation, and thus the presence of these MMPs is usually regarded as one of the marks of functional mature invadopodia. In addition, the potency of invadopodia to degrade ECM and facilitate invasion is partially attributed to MMPs appearance [79, 80].

The ECM serves as an indispensable reservoir for many growth factors and cytokines that orchestrate diverse developmental processes and can trigger a series of signal transduction to induce sustained malignant transformation [59, 81]. As such, the degradation of ECM also contributes to tumor development by these secretory factors. For example, transforming growth factor-β (TGF-β), an essential cytokine for the activation of tumor stroma, is significantly overexpressed in the dysregulation ECM and induces immunosuppression within the TME [82]. The release of VEGF is sometimes accompanied by the remodeling ECM and further contributes to angiogenesis [83]. Hepatocyte growth factor (HGF) is a pleiotropic cytokine. Mature HGF retained in the ECM is able to bind its receptor c-MET to mediate cancer progression [84]. Furthermore, Oncostatin M (OSM), a proinflammatory cytokine, was demonstrated to induce the expression of lysyl oxidase like-2 (LOXL2) that catalyzed ECM transformation by crosslinking collagen I. The overexpressed OSM and LOXL2 had an evident correlation with a worse prognosis in patients with breast invasive ductal carcinoma [85]. The dynamic ECM also promotes the presentation of growth factors to their receptors [86, 87].

### Cancer-associated vasculature

During malignant transformation, tumor tissues establish sophisticated compositions to support their growth. These compositions include an immunosuppressive TME, a nutritional environment suitable for tumor growth, and the unique cancer-associated vascular system. Angiogenesis is central to the growth and survival of tumor cells and is also the main conduit for tumor metastasis [88]. Approximately five decades ago, Folkman described that neovascularization promoted tumorigenesis and malignant progression. He held the view that destroying tumor angiogenesis could restrict nutrient supplies to malignancies and speculated that anti-angiogenic drugs would have potential therapeutic value for cancer [89].

Tumor tissue’s vascularization is a multidimensional process orchestrated by various molecular and cellular effectors [90]. Compared with normal stroma, the tumor stroma has abundant vasculature. Pancreatic ductal adenocarcinoma (PDAC) represents one of the most stroma-rich cancer types. Cancer-associated vasculature constitutes an integral part of the stroma in PDAC [91]. Clinically, the intratumoral microvessel density (MVD) was associated with adverse prognosis and could be regarded as an independent prognostic factor [92, 93].

The initial step of angiogenesis usually involves the action of diverse angiogenic stimuli such as hypoxia [94]. Under hypoxic conditions, tumor cells constantly consume glucose and then secrete lactate to create an acidic stromal environment that favors angiogenesis. Hypoxia-inducible factor (HIF) has a pivotal role in the responses of tumor cells and stromal cells to hypoxia [95]. HIF-1 was upregulated within a hypoxic environment, which further resulted in increased expression of VEGF and positively affected cancer metastasis [96, 97]. The downregulation of HIF-1α through the CRISPR/Cas9 technique was found to dramatically inhibited the migration of BxPC-3 cells achieved by decreased expression of MMP-9 and VEGF [98]. Therapeutic modalities based on anti-VEGF can repress human PDAC cells’ growth in murine models and reduce microvessel density, ultimately leading to depleted tumor angiogenesis [12].

A wide spectrum of pro-angiogenic factors and related cognate receptors partake in the activation of “angiogenic switch” and the formation of tumor vasculature [99]. Among all pro-angiogenic factors, VEGFs represent one of the most potent angiogenesis inducers and function by binding to their specific receptors VEGFR or co-receptors. VEGFA is the key angiogenesis regulator and the most investigated member of VEGF family [100]. Other key secretory factors involved in abnormal angiogenesis include fibroblast growth factor (FGF), platelet-derived growth factor (PDGF), HGF and angiopoietins [88]. Furthermore, several chemokine signaling axes also contribute to tumor vasculature generation such as CXCLs/CXCR2, SDF1/CXCR4, and CCL2/CCR2 axis directly or indirectly [88].

Cancer-associated vasculature not only provides nutritional supplies for tumor tissues but also accelerates the transformation of pre-malignant to malignant and aggressive tumor phenotypes. Continuous vascular remodeling is an important characteristic of the established microvasculature of growing tumors. During cancer progression, host vasculature can be used as trails for the invasion of glioma cells into adjacent tissues, which make tumor cells acquire an aggressive character [101].

Taken as a whole, while the stromal composition vary across distinct cancer types, some major components are indispensable for solid tumors, especially the cancer-associated vasculature that has been shown to promote tumor growth and mediate the invasive tumor phenotype [23, 102]. All the above preclinical studies indicate that targeting stromal vasculature may be an effective tactic for cancer treatment. Unfortunately, the use of anti-angiogenic drugs to treat cancer patients often shows limited benefit and even has been a clinical failure, which poses a significant challenge in terms of how best to design this therapeutic option to ultimately elicit an efficacious antitumor response [99].

### Tumor-associated endothelial cells

Recent studies have identified the central roles of tumor-associated endothelial cells (TECs) in instigating cancer initiation and progression. TECs usually exhibit phenotypes distinct from normal endothelial cells, because they are aneuploid and their centrosomes are abnormal [103]. Functionally, TECs actively promote the proliferative and aggressive capacity of cancer cells, as well as induce resistance to anti-tumor agents [104, 105].

TECs acquire high proliferative and invasive abilities and accelerate tumor cell growth by secreting soluble factors in a paracrine manner [106, 107]. Furthermore, TECs support malignant cell aggressive behavior by mediating the epigenetic dysregulation of secreted molecules and activating metastasis-associated signaling circuits [108, 109]. Activated TECs can also be released in the blood circulation system from the primary tumor mass and accompany with cancer cells to migrate to distant secondary sites [110]. Compared with low-metastatic tumor-derived TECs, those high-metastatic TECs possess higher mRNA expression level of stemness-related gene stem cell antigen and mesenchymal marker CD90, as well as higher levels of vascular secretion factors [111].

TECs mediate drug resistance and convert naive cancer cells into chemoresistant tumor stem-like cells [112, 113]. For a long time, TECs have been recognized as a group of normal diploid cells that would not induce therapeutic resistance. However, mounting researches found that TECs usually showed aneuploidy and had hallmarks of chromosomal instability, which may contribute to therapeutic resistance in antitumor treatment [103, 114, 115]. Indeed, scientists unveiled their drug resistance ability in different cancer types, such as renal carcinoma-derived TECs inducing vincristine resistance and hepatocellular carcinoma-derived TECs resistance to adriamycin, 5-fluorouracil and sorafenib [115–117]. It was also elucidated that TECs could upregulate the expression of p-glycoprotein (p-gp), one of the ABC transporters, to impair antitumor therapy. Inhibition of p-gp with verapamil abrogated TECs resistance, restored the chemosensitivity of tumor cell to paclitaxel and depleted tumor angiogenesis in the mouse model [118, 119].

Altogether, in addition to promoting angiogenesis, TECs serve as key players to partake in various steps of malignant transformation, and this is a relatively unexplored field that can potentially provide crucial insights into tumor progression.

### Cancer-associated fibroblasts

**Fig. 2:**
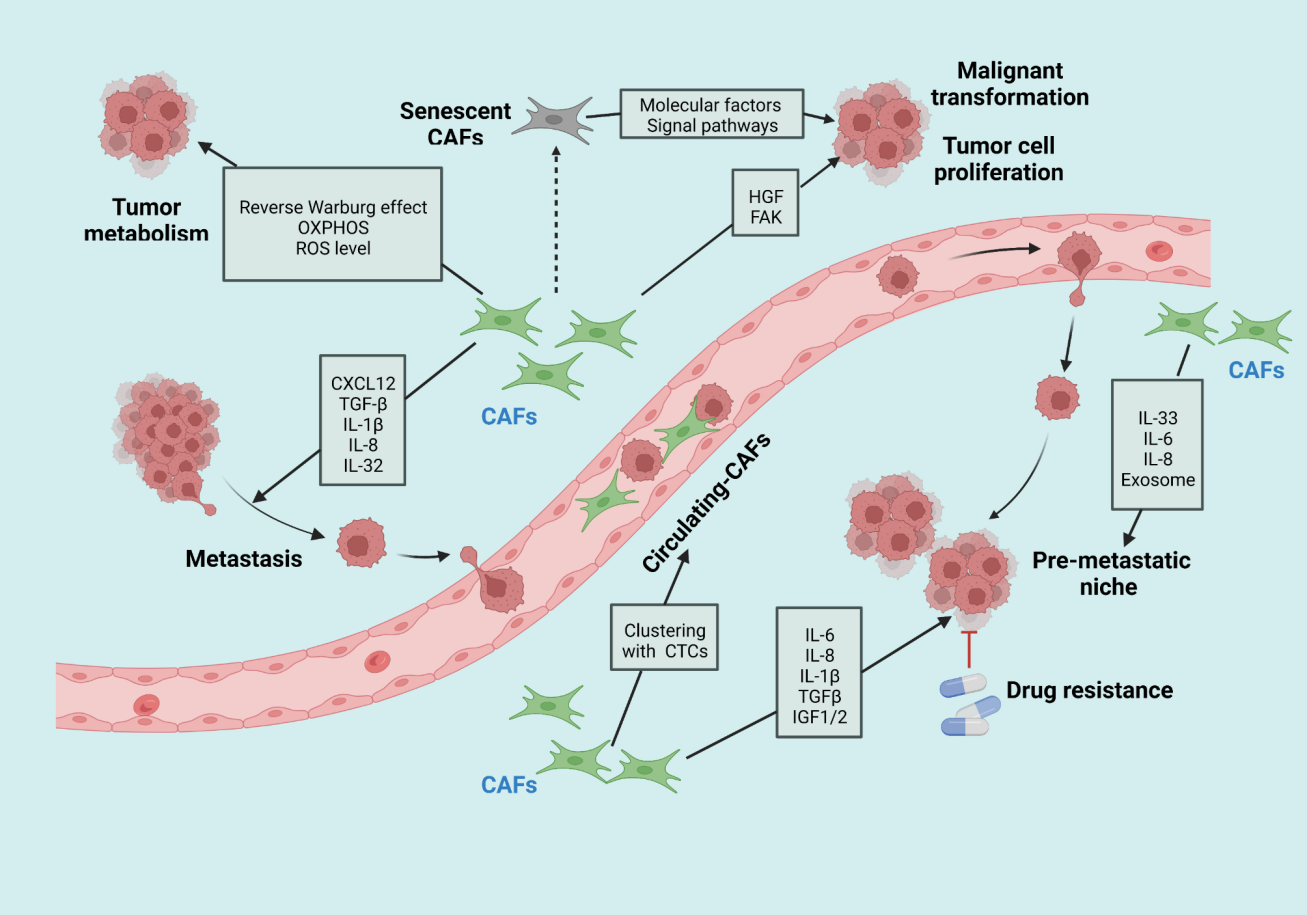
A brief summary of CAFs functions and related mechanisms in cancer initiation and progression. Activated CAFs are involved in nearly all stadges of cancer development through diverse means. By releasing numerous secretory factors and activating signaling pathways, CAFs contribute to maliganant transformantion, tumor growth and proliferation, cancer cell invasion, and the establishment of pre-metaststic niche. These pro-tumorigenic cells also affect tumor metabolism in distinct manners as shown in the figure. All these functions collectively determain tumor development and drug resistance

Among all stromal cellular components, cancer-associated fibroblasts (CAFs) are one of the prominent and abundant cell populations. Activated fibroblasts found in primary or metastatic tumors are referred to as CAFs. They provide a physical support for cancerous cells and affect cancer initiation, progression and metastasis (Fig. 2) [120–122]. Of note, CAFs exert both protumorigenic and antitumorigenic effects during disparate stages of oncogenic advancement in an organ or context-specific manner, which brings challenges to the area of CAFs-targeted therapy for cancer treatment [123, 124].

CAFs tend to exhibit heterogeneity and complexity with distinct origins, cellular states and functions. In contrast to normal fibroblasts, CAFs have been described as proliferative, migratory, and highly secretory cells [125]. Although it is generally accepted that most CAFs possibly originate from the activation of local tissue-resident fibroblasts [126], researchers have also identified other major cellular origins of CAFs including endothelial cells [127], adipocytes [128], bone marrow-derived mesenchymal stem cells [129–131], and pancreatic or hepatic stellate cells [132, 133]. Hence, it is difficult to precisely define where these cells originate from.

After being activated by diverse signaling pathways, CAFs derived from various cellular precursors exert many of their functions in cancer [134]. More recently, Silvia Affo et al. discovered that HGF produced by CAFs together with its receptor MET highly expressed in cancer cells instigated the proliferative activity of intrahepatic cholangiocarcinoma (ICC) tumor cells, which is primarily associated with the ERK and AKT phosphorylation. Furthermore, hyaluronan synthase 2 derived from CAFs, but not type I collagen, could effectually regulate the promoted effect on ICC [135]. Intriguingly, tumor cells not only attract fibroblasts and transform them into CAFs but can also be regulated by CAFs to sustain their proliferation and migration [136]. Cancer cell usually undergo metabolic reprogramming during tumorigenesis that can be modulated by CAFs [137]. As opposed to normal cells, tumor cell utilize glycolysis as their preferred energy source, which is often accompanied by increased production of lactate, known as the “Warburg effect” [138, 139]. Recently, Pavlides et al. proposed a novel hypothesis termed as “reverse Warburg effect” that might represent a general feature of CAFs. Tumor cells induce the Warburg effect in adjacent stromal CAFs. Then, in response to changes in the TME, these CAFs secrete pyruvate and lactate that can be used by epithelial cancerous cells to generate energy and enhance their proliferative capacity via oxidative phosphorylation (OXPHOS) [140]. The metabolism of tumor cells is also influenced by CAFs-modulated autophagy and oxidative stress pathway to promote tumor cell proliferation and drug resistance [141–145].

CAFs mediate the invasion and migration of malignant cell and are positively associated with the dedifferentiation and aggressiveness of cancers [136]. Recently, four CAFs subsets, named CAF-S1 to -S4, were identified in metastatic lymph nodes (LNs) of breast cancer (BC). Among them, both CAF-S1 and CAF-S4 subpopulations could be preferentially detected in tumor tissues and were proven to be closely related to tumor cell invasion in a complementary manner [146, 147]. CAF-S1 stimulated BC cell motility and epithelial-mesenchymal transition (EMT) initiation via CXCL12/TGF-β signal whereas CAF-S4 remodeled the matrix and promoted BC cell invasiveness in 3-dimensions via NOTCH-mediated pathways [147]. Currently, our studies about the function of CAFs subsets mainly converge on CAF-S1. In addition, the enrichment of CAF-S1 was correlated positively with PD-1^+^ and CTLA-4^+^ CD4^+^ T cell content but negatively with CD8^+^ T cells infiltration in tumor. CAF-S1 subset also can enhance the expression of PD-1 and CTLA-4 at the surface of CD4^+^ CD25^+^ FOXP3^+^ T lymphocytes to participate in the formation of immunosuppressive environment within tumor mass[148]. Clinically, the enrichment of CAF-S1 in stroma was significantly correlated with cancer recurrence [149]. Additionally, CAF-S3 subset was mainly detected in juxta-tumors whereas CAF-S2 equally distributed between the tumor mass and juxta-tumors [146]. While the distribution of CAF-S2 and CAF-S3 in tumors have been reported, their specific effects of CAF-S2 and CAF-S3 in tumorigenicity remain to be fully characterized and identified.

Secretory proteins derived from CAFs also partake in tumor cells invasion in an autocrine or paracrine manner. For instance, Lumican, an ECM protein expressed in human gastric CAFs, was found to have promoting effect on GC cells growth and migration in vitro by activating the integrin β1-FAK signal [150]. A great variety of soluble paracrine growth factors, cytokines, and exosomes secreted by CAFs also profoundly impacted malignant cells migratory and aggressive capacity in established tumors. These factors comprise, but are not restricted to, interleukin‑1β (IL-1β), IL-8, IL-32, CXCL12, and TGF-β [151–156].

In metastatic process, the pre-metastatic niche (PMN) acts as a fertile “soil” that supports the homing and engraftment of circulating tumor cells. CAFs are actively involved in the formation of PMN [157, 158]. CAFs-derived IL-33 was responsible for establishing the PMN in lung that facilitated pulmonary metastasis of breast cancer. This promoting effect was associated with type-2 inflammation and the recruitment of diverse immune cells such as eosinophils, neutrophils and inflammatory monocyte to the lung microenvironment [159]. Likewise, in the lung metastatic niche, high-metastatic hepatocellular carcinoma cells typically exhibited great ability to convert normal fibroblasts into CAFs, which was mediated by exosomal miR-1247-3p derived from HCC cells that activated CAFs via the B4GALT3-β1-integrin-NF-κB axis. And then, activated CAFs further accelerated cancer cells diffusion and metastasis by secreting pro-inflammatory IL-6 and IL-8 [160].

Drug resistance remains one of the major hurdles in cancer management. Stromal CAFs have been associated with resistance to anticancer agents by secreting numerous proteins, cytokines and extracellular vesicles. These factors can activate different signaling cascades to protect cancer cells from elimination and possibly cause recurrence [161, 162]. High levels of IL-8 released by CAFs have been identified to be associated with poor response to neoadjuvant chemotherapy. Mechanistically, IL-8-mediated resistance to cisplatin was achieved by NF-κB activation and ATP-binding cassette subfamily B member 1 upregulation [163]. CAFs-derived exosomal miR-98-5p was reported to suppress ovarian cancer (OC) cells apoptosis and promote their proliferative capacity by targeting cyclin-dependent kinase inhibitor 1 A that contributed to the sensitivity of OC cells to cisplatin [164]. Another secretome makes contribution to chemotherapeutic resistance, such as IL-1β [165], IL-6 [166], insulin-like growth factors (IGF) 1 and 2 [167], TGF-β [168], etc. Radiation therapy also leads to expansion and survival of stromal CAFs, which conversely provides signals stimulating malignant cell proliferation and enhances radioresistance.This radioresistance involved various mechanisms including paracrine IGF-1/IGF-1R signaling initiated by CAFs, signaling transduction regulated by exosomal miRNAs or exosomes derived from CAFs, and increased level of ROS mediated by CAFs-derived molecules [169–172].

Circulating-CAFs (cCAFs), similar to circulating tumor cells (CTCs), can be detected in approximately 88% of patients with metastatic breast cancer (BC) and 23% of patients with localized BC. These circulating-CAFs exist as homotypic cCAF clusters individually or present as heterotypic clusters together with CTCs and leukocytes [173]. Sharma et al. unveiled that CD44 acted as an indispensable mediator in cCAF-CTC heterotypic clustering and the cCAF-CTC clusters existed in nearly all clinical stages of BC [174]. Circulating fibroblast-like cells were also detected in blood of metastatic prostate cancer patients and could potentially serve as a prognostic marker [175].

Senescence is another characteristic of CAFs. Senescent CAFs usually acquire tumor-promoting properties, termed as senescence-associated secretory phenotype, which promotes malignant transformation by secreting molecular factors or driving downstream signal pathways [176]. By activating JAK/STAT3 signaling, senescent CAFs (s-CAFs) enhanced GC cells proliferative activity and contributed to peritoneal tumor formation of GC in vivo [177]. Specific induction of s-CAFs apoptosis remarkably enhanced radiosensitivity of non-small cell lung cancer cells [178].

To date, studies on the role of CAFs in cancer progression have gained momentum with increasing attention. Our understanding of their definitive functions in various cancer types will quickly evolve in the near future. The continuous exploration of new pro-tumorigenic molecular mechanisms for CAFs is likely to have profound implications for anticancer therapy.

### Mesenchymal stromal cells (MSCs)

**Fig. 3:**
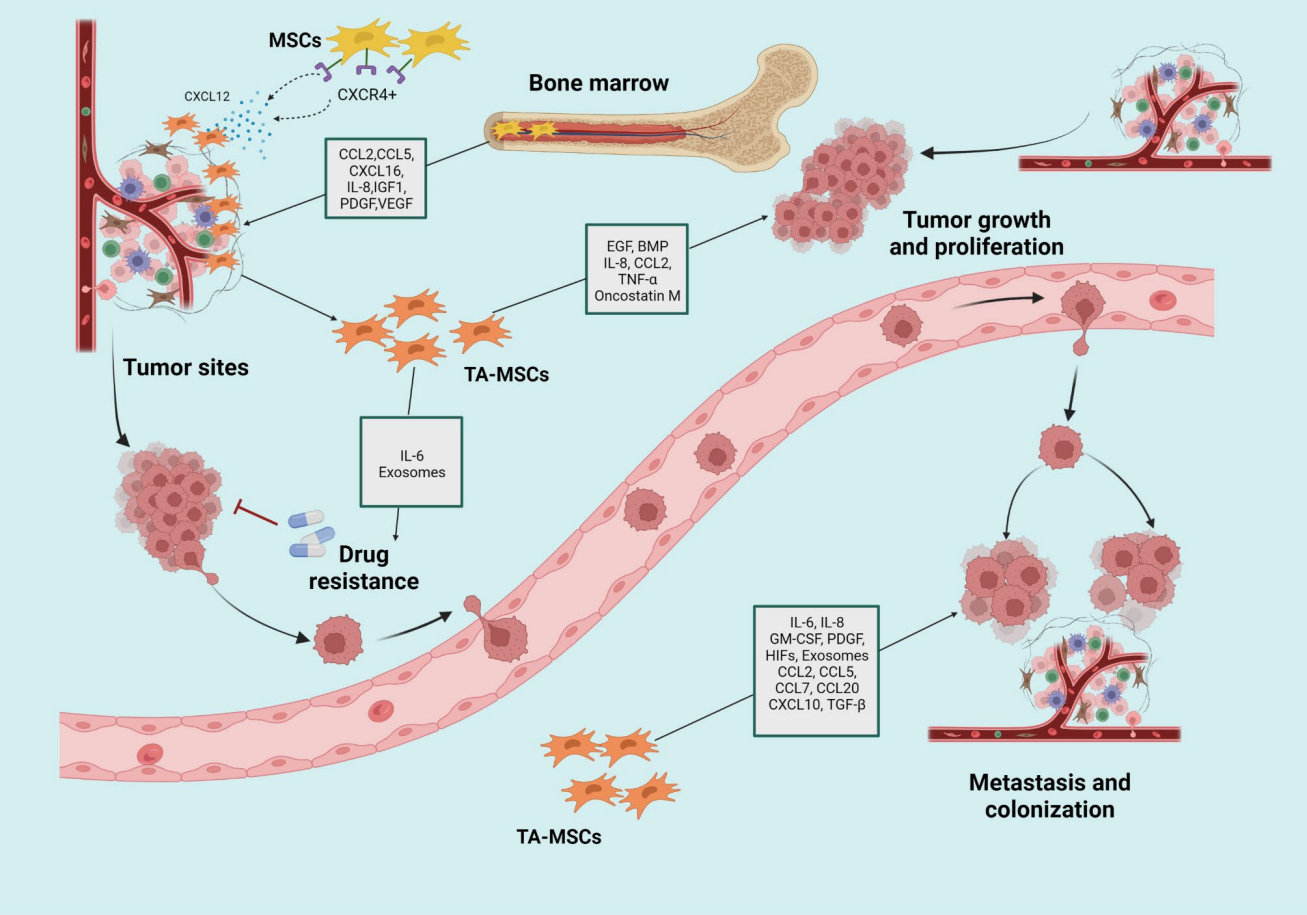
The tumor-promoting functions and related mechanisms of MSCs and TA-MSCs

Mesenchymal stromal cells (MSCs) represent a group of pluripotent nonhematopoietic stem cells that have self-renewal ability and play substantial roles in tissue regeneration. MSCs have capacity to differentiate into osteoblasts, chondrocytes, or adipocytes in culture and then perform their different functions depending on the circumstances [179, 180]. Notably, MSCs can migrate to tumor tissue where they further evolve into tumor­associated MSCs (TA­MSCs) that probably are distinct from those of normal tissue MSCs and have a pro-tumorigenic phenotype [181, 182] (Fig. 3).

The recruitment and migration of MSCs to tumor sites are affected by chemokines and growth factors, which in turn promotes cancerous development. The CXCL12/CXCR4 axis represents one of the most intensively studied pathways in the tumor tropism of MSCs [183, 184]. Tumor conditioned medium could upregulate CXCL12 expression that facilitated the migration of human BM-MSCs to tumor sites by activating JAK2/STAT3 and MEK/ERK1/2 pathways [185]. The capacity of MSCs to move toward tumor tissue is also mediated by other factors, such as chemokines CCL2, CCL5, CXCL16 [186–188], diffusible cytokine IL-8, as well as growth factors IGF1, PDGF, VEGF, and TGF-β [189, 190].

Compared with normal MSCs, TA-MSCs appear to transform into an “activated” state and undergo epigenetic reprogramming modulated by the TME. This reprogramming mediates a partial mesenchymal-to-epithelial transition that results in enhanced binding of TA-MSCs to cancer cells, thereby effectively favoring the colonization of TA-MSCs and tumor cell complex in metastatic sites [191]. In a mouse lymphoma model, following co-culturing with TA-MSCs, BM-MSCs acquire a tumor­promoting phenotype that depends on the recruitment of macrophages to tumor sites mediated by CCR2 [192]. However, the ability to become TA-MSCs might depend on a particular tissue or cancer type because the observation that breast cancer TME could reprogram BM-MSCs into TA-MSCs that dramatically promoted cancer cells growth whereas the ovarian TME could not [193].

After reaching at the tumor niche, TA-MSCs influence tumor development through direct and indirect manners. The direct cell-to-cell interplay between co-injected MSCs and MDA-MB-231 significantly increased intratumoral cancer cells viability and promoted their proliferation [194]. Following isolating and identifying from the primary tumor tissue, TA-MSCs manifested the homogenous immunophenotype and were shown to have differentiation potential. Furthermore, TA-MSCs could secrete epidermal growth factor (EGF) which activated the downstream PI3K/AKT signaling to modulate cancer cells proliferative activity [195]. TA-MSCs also activate neutrophils with enhanced expression of IL-8, CCL2, TNFα, and oncostatin M, as well as affect the chemotaxis of neutrophils to protect them from spontaneous apoptosis. TA-MSCs-educated neutrophils promote GC cells growth and migration in a cell contact-dependent manner [196]. Moreover, TA-MSCs were determined to facilitate tumor cell proliferation by increasing cancer stem cell numbers and augmenting BMP production [197]. Except for assisting malignantly transformed cell growth as mentioned above, TA-MSCs help cancer cells defend against senescence via the P53/P21 pathway and then prolong their survival cycle [198].

TA-MSCs also play critical roles in creating a favorable condition for successful metastasis of tumor cells. After being isolated from human colon cancer, TA-MSCs were shown to dramatically enhance the invasive activity of HCT-116 cells in vitro. IL-6 existing in the TA-MSCs-conditioned medium induced the enhanced surface expression level of CD44 in HCT-116 and HT-29 cell lines via Notch signaling to promote colon cancer progression [199]. On the basis of the initial inspection that visible tumor metastasis occurred in the tumor cell plus TA-MSCs group rather than the tumor cell-only group, Waghray et al. found that GM-CSF was the only cytokine secreted by the TA-MSCs in all tested patient samples and it can induce tumor cells EMT to drive metastasis [200]. Analogously, TA-MSCs contributed toward the M2 polarization of macrophages, which further significantly augmented the EMT process of GC cells [201]. The mutual transcriptome modulation between MSCs and tumor cells also impacts metastasis process. Tumor cells along with stromal factors partake in promoting normal MSCs conversion toward TA-MSCs, and in turn, TA-MSCs cause the upregulation of tumor metastasis-associated genes in primary lung cancer cells and selectively foster their migration and dissemination [202].

After being stimulated by cancer cells, TA-MSCs can produce the chemokine CCL5, which in turn acts on tumor cells in a paracrine manner to induce their motility and metastatic ability [203]. Other chemokines or cytokines secreted by TA-MSCs have also been shown to drive metastasis, such as CCL2, CCL7, CCL20, CXCL10, TGF-β, PDGF, IL-8, IL-6, and HIFs [204–212]. They collectively act as an extra driving force to support the successful dissemination of tumor cells from primary mass to metastatic sites. Furthermore, TA-MSCs-derived exosomes facilitated cancer cell growth and migration as well as potentially could served as biomarkers for GC [213]. Some scientists have suggested that TA-MSCs play a role in preparing PMNs for cancer cells, but the underlying mechanisms are not fully understood and merit further study for years to come [190].

TA-MSCs render tumor cell resistant to chemotherapy. In addition to sustaining tumor cells growth and assisting their metastatic ability, IL-6 was also reported to reduce cisplatin-triggered apoptosis in breast cancer cells via the STAT3 pathway [214]. The drug resistance effect and stemness of cancer cell is also expedited by TA-MSCs mediated-LncRNA secretion. There are reports suggesting that MSC-associated MSC-AS1 and AGAP2-AS1 mediate drug resistance through the PI3K/Akt signaling pathway and regulation of CPT1 expression, respectively [215, 216]. Targeting these MSCs or suppressing the cytokines and LncRNA expression they adjust may be a optimal approach to resensitize the tumors to anticancer therapy.

In short, TA-MSCs perform their tumor-promoting properties by sustaining tumor cell growth and proliferation, altering tumor cell phenotype and conferring them an aggressive or migratory ability, decreasing treatment response to various drugs, and even preparing a pre-metastatic niche for circulating tumor cells. Accumulating evidence unraveling the role of TA-MSCs in multiple cancer types at different steps of tumor development provides a novel insight for us to understand the important function of stroma in cancer.

### Pericytes

Long known as regulators of vascular morphogenesis and function, pericytes represent a cell type neighboring the microvascular periendothelial mesenchyme and have been reported to participate in multiple pathological processes, especially malignant tumors [217]. Pericytes can interact with tumor cells or stromal cells to alter the TME and exert immunomodulatory functions, thereby contributing to tumorigenic processes and metastatic dissemination [218, 219]. Interestingly, tumor cells have potential to generate pericytes, since the observation that, in glioblastoma xenografts, approximately 89% of vascular pericytes originate from glioblastoma stem cells [220].

It has been shown that a pericyte co-culture system promoted ovarian cancer cells’ growth and invasion in vitro. In the xenograft model, tumor cell OVCAR-5 or OVCAR-8 along with pericytes were co-injected into nude mice, which resulted in accelerated tumor growth and invasive metastasis compared with injection of cancer cell alone, without altering and affecting tumor vasculature. The high pericyte score was highly predictive for poor prognosis of cancer patients [221]. Some secreted factors from pericyte were involved in oncogenic development. For example, pericytes secreted insulin-like growth factor 2 was found to have a pro-proliferative effect on breast cancer cell and contributed toward the formation of brain metastasis [222]. It was also found that deletion of β3 integrin in pericytes accelerated primary tumor growth and exacerbated cancer progression without influencing tumoral angiogenesis [223]. As for the pro-metastatic effects, preparing a PMN for cancer cells is a key section mediated by pericytes [224]. Furthermore, pericyte can be induced by PDGF-BB to transdifferentiate into fibroblast, which is important to facilitate tumor metastasis and offers a novel targeted option for anti-metastasis therapy [225].

Even though these valuable results have been obtained, it should be noted that our current comprehension regarding the concrete roles of pericytes in cancer is still relatively insufficient, and additional exploration is also warranted.

## Interplay and crosstalk between intrastromal components

**Fig. 4:**
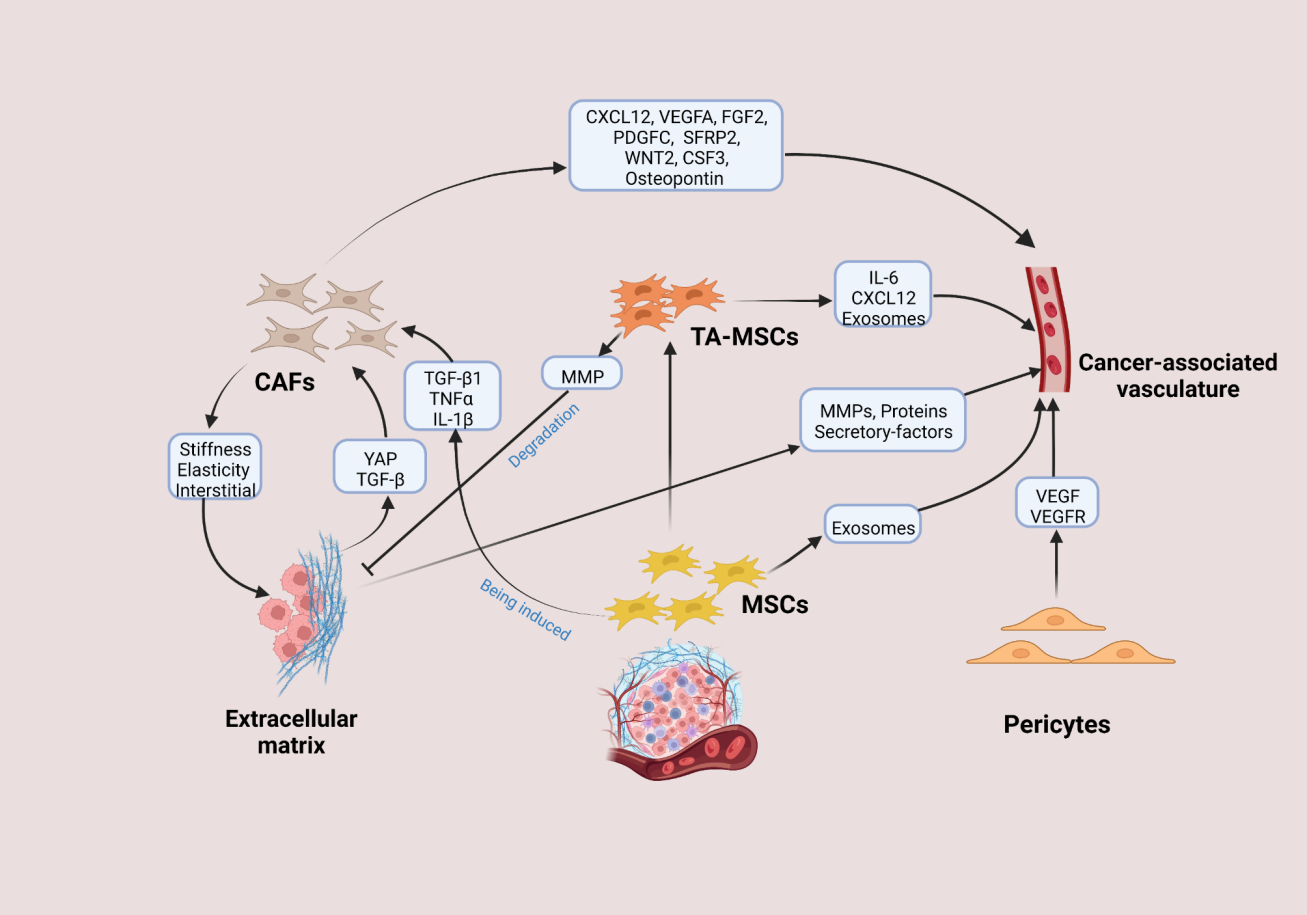
Interactions between stromal elements and each others

The tumor stroma is not a quiescent entity. Instead, it is a highly dynamic and constantly changing environment with complex elements that can interact with cancer cells to affect tumor behavior. Apart from directly interplaying with malignancies, the stromal components work in concert with one another, presumably impacting the unrestricted growth, invasion and propagation of a tumor through the body. The intrastromal crosstalk orchestrates multiple biological processes (Fig. 4), and a better understanding of their reciprocities is expected to shed substantial light on the investigation of tumor stroma and their roles in cancer.

### Interaction between stromal cellular elements and angiogenesis

During malignant transformation, tumor cells acquire a capacity to reshape and educate surrounding stroma to meet their nutrient requirements, which eventually induces unremitting angiogenesis. Simultaneously, the mobilization and activation of stromal cells and the infiltration of capillaries into tumor tissues are thought to be a prerequisite for tumor growth and metastasis. Because ECM usually serves as an essential repository of diverse effector molecules, it is not surprising that ECM profoundly impacts on the formation of cancer-associated vasculature. Following being affected by a series of pro-angiogenic signals, endothelial cells tend to migrate into the interstitial matrix and release MMP that can remodel the basement membrane surrounding the vasculature. Moreover, considerable tenascin, fibronectin, remodeled type I and III collagens existing in ECM stimulate angiogenesis [37, 226]. The stiffened ECM also drives angiogenesis by facilitating the activation of splicing factors to enhance the production of PKC βII and VEGF 165b alternative splice variant in endothelial cells [227]. The ECM also contributes to the expression of VEGFR and its internalization in endothelial cells, which helps endothelial cells survive by sustaining the ERK signaling [228].

The stimulative effects of CAFs on neovascularization are principally achieved by many secretory pro-angiogenic factors including CXCL12, WNT2, VEGFA, FGF2, PDGFC, secreted frizzled-related protein 2 (SFRP2), CSF3, and osteopontin [90, 229–231]. Also, CAFs can indirectly attune tumor vascularization via the biomechanical modulation of ECM stiffness, elasticity and interstitial fluid pressure [90, 232, 233]. Analogously, extensive studies regarding the role of TA-MSCs in building cancer-associated vasculature largely concentrate on pro-angiogenic chemokines or growth factors such as VEGF, IL-6, and the CXCL12/CXCR4 axis [234–236]. It has also been demonstrated that TA-MSCs drive angiogenesis through transdifferentiation into endothelial cells or the recruitment of endothelial progenitors [190]. Furthermore, MSCs can release exosomes that transfer miRNA to endothelial cells and contribute to angiogenesis in vitro, but whether the semblable result exists in the context of tumor deserves to further verify [237]. The recruitment of pericytes is indispensable for vasculature formation and maturation, since they can interact with endothelial cells to stimulate basement membrane matrix assembly, relay growth factors such as VEGF to modulate the survival of endothelial cells, and respond to VEGF by expressing VEGFR1 [238, 239].

### Interaction between CAFs, the ECM and MSCs

Nowadays, mounting evidence has linked CAFs with the tumor ECM. On the one hand, CAFs may be the most effective cell type in building up and remodeling the structure of ECM, which is partially attributed to their ability of assisting tumor cells to migrate through the stroma and interact with other stromal elements. CAFs can synthesize and release many ECM proteins including collagens, laminin and fibronectin. Moreover, matrix-crosslinking enzymes produced by CAFs along with force-mediated ECM reconstitution are responsible for the enhancive stiffness of tumor tissues [121, 126]. On the other hand, the activation of CAFs is affected by some physical changes in the ECM. For example, one of the signature features of CAFs is to activate YAP transcription factor required for CAFs to induce increased matrix stiffness, and intriguingly, stiffened ECM in turn sustains CAFs phenotype by promoting the activation of YAP [240]. Additionally, the convergence of both ECM composition and elasticity together with TGF-β can influence the phenotypic heterogeneity of CAFs, which has potential value for further development of stroma-targeted treatment [241]. It has also been implied that TA-MSCs are capable of producing MMPs and then degrading ECM to impact the configuration of pro-metastatic tumor ECM [190].

During the interplay with cancer cells, MSCs can be induced to differentiate into CAFs. In the setting of prolonged exposure to cancer-conditioned medium, human MSCs could possess up-regulation of CAFs-associated genes and display functional properties of CAFs characterized by consistent expression of SDF-1 and higher expressed levels of α-SMA, vimentin, and fibroblast surface protein [131]. Specifically, the mobilization of MSCs to tumor sites and the transdifferentiation of MSCs into CAF-like cells are partially mediated by TGF-β1 derived from both cancer cells and tumor-educated-stromal cells [242]. Additionally, under the sustained stimulation with pro-inflammatory cytokines TNFα and IL-1β, MSCs converted into CAFs, and importantly, these CAFs release diverse factors to stimulate CCR2, CCR5, CXCR1/2 and Ras-activating receptors existing in cancer cell surfaces, thereby enhancing cancer cell dispersion and metastasis [243].

## Stromal elements and the immune system

**Fig. 5:**
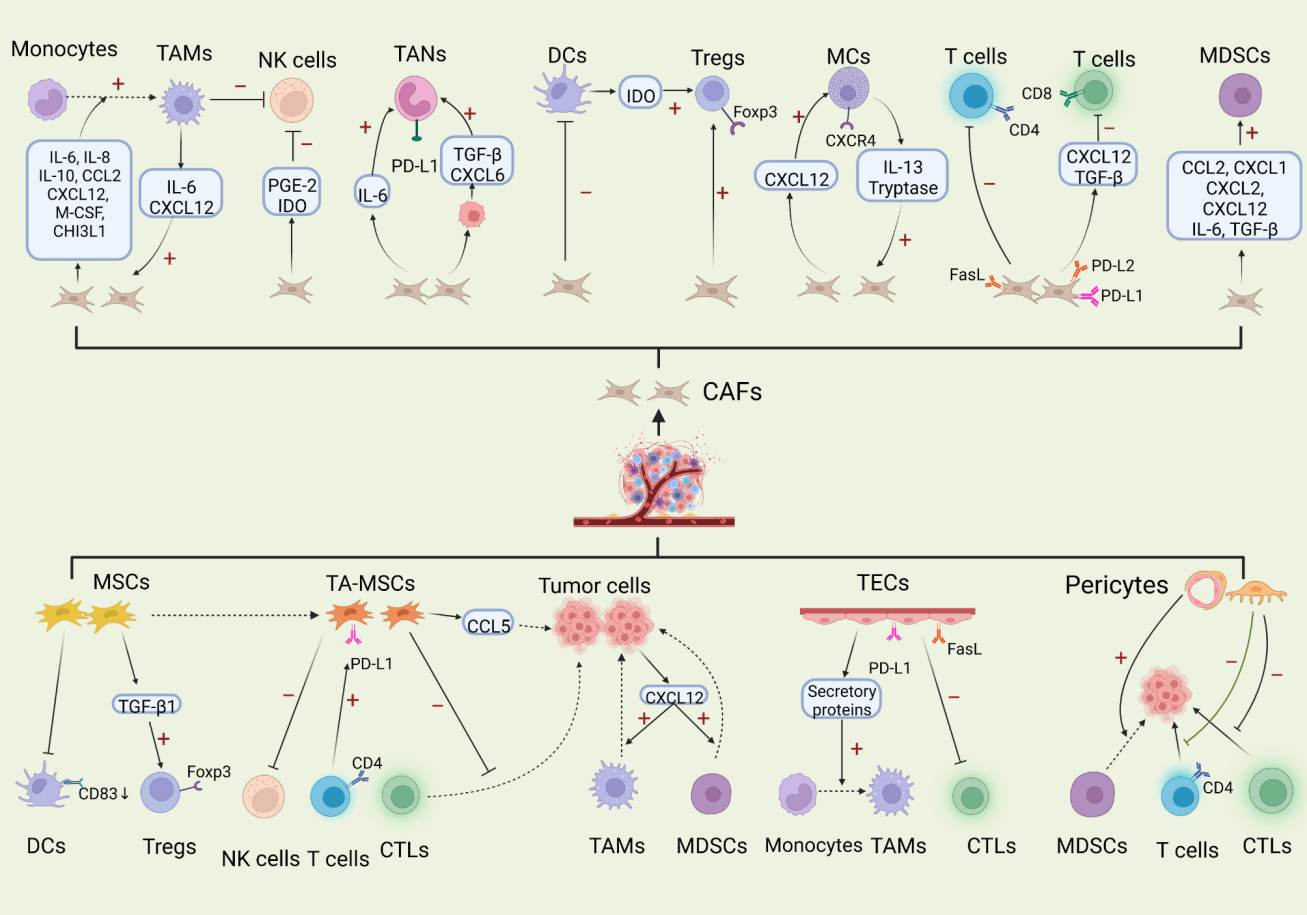
Interactions between stromal cells and diverse immune cells

The immune system is typically thought to be a master mediator for cancer and plays crucial roles throughout the tumor initiation and progression. Arguably, immune cells exist in large quantities in the TME and attune the body’s response to malignant tumors. Most of stromal elements, if not all, jointly contribute toward forming of an immunosuppressive TME that enables cancer cells to evade surveillance and attack from body’s immune system (Fig. 5) [244].

### The ECM and the immune system

It has been illustrated that the ECM participated in modulating the differentiation, migration, infiltration and polarization of immune cells residing in the TME, and therefore supporting or compromising antitumor immunity. The ECM not only provides crucial migratory cues for immune cells but also also serves to affect their function [245–247]. Loose regions of fibronectin and collagen assist T cell motility and migration in chemokine-dependent ways, whereas dense ECM areas impede T cell trafficking and lead to reduced number of infiltrating CD8^+^ T-cells, suggesting that thickened ECM interferes with antitumor responses by governing the motility and positioning of T cell [248–250]. Furthermore, a recent study uncovered that interfering with collagen stabilization could deplete the content and stiffness of ECM, resulting in increased efficacy of anti-PD-1 therapy and effective T cell infiltration [251]. As for the contribution of ECM to immune cell’s function, an important aspect is their repressive role on T cell. In regard to this, stiffened ECM can impair the antigen presentation by APCs and decrease the production of IL-2 that is responsible for promoting Th1 cells’ differentiation and T cell’s proliferation [41, 252]. Furthermore, the ECM protein Tenascin-C can interact with α5β1 integrin on the T cell surface to impair reorganization of the actin-based cytoskeleton that is necessary for T cell activation [253].

Tumor areas that exhibit the highest levels of collagen cross-linking tend to demonstrate ample macrophage infiltration in the condition of breast cancer. Therapeutic ablation of these accumulated macrophages can reduce metastasis and stromal stiffening, which indicated that collagen cross-linking likely contributed to the recruitment of macrophages and drove tumor metastasis [254]. The tumor ECM also favors the infiltration of macrophages within tumor tissue and drives their polarization to M2-like phenotype to exert immunosuppressive function [255–257].

### Cancer-associated vasculature and the immune system

Cancer-associated vasculature not only provides nutrient supply for tumor growth but also impedes effective drug delivery to tumor sites sometimes because of its abnormal structure. Importantly, tumor vasculature contributes to the formation of an immunosuppressive TME by limiting entry of effector T cells [258]. Also, hypoxic surroundings within a tumor caused by abnormal blood perfusion can accelerate the differentiation of tumor-infiltrating myeloid cells to M2-like tumor-associated macrophages (TAMs)[259, 260]. Meanwhile, hypoxia also supports the differentiation and function of MDSCs and Tregs via various immunosuppressive molecules to mediate antitumor immune escape [261]. Combination of vasculature targeting and immune checkpoint inhibitor was demonstrate to elicit potent antitumor response in preclinical study, which endow the further application of inhibiting vasculature plus immunotherapy high promise [262].

### TECs and the immune system

TECs are responsible for protecting tumor cells from the host immune attack[263]. TECs-derived secreted protein mediated the M2 polarization of macrophages by activating the PI3K/AKT/mTOR pathway [264]. Notably, TECs can express the death mediator Fas ligand following the cooperatively inducing by several factors including VEGF-A, IL-10, and prostaglandin E2 (PGE2), thus obtaining the ability to kill effector CD8^+^ T cells rather than regulatory T cells (Treg) to enhance tumor cell escape [265]. TECs also induce CD8^+^ T cell infiltration and exhaustion via the expression of glycoprotein nonmetastatic melanoma protein B in hepatocellular carcinoma [266]. Moreover, TECs tend to exhibit elevated PD-L1 phenotype, so as to bind to programmed death 1(PD-1) in activated lymphocytes and hinder the body’s immune response [267, 268].

### Activated CAFs and the immune system

Activated CAFs play structural and functional roles within the immune system through diverse manners including remodeling the ECM to create a physical immune barrier, regulating the antitumor activity of tumor-infiltrating immune cells, and facilitating the expression level of immune checkpoint molecules [269–271].

In the innate immune response, TAMs are perhaps the most predominant cells neighboring CAF-populated areas and have multidimensional interactions with CAFs. CAFs actively promote the recruitment of monocytes into tumor areas where they further evolve into the protumorigenic M2 macrophage subset [272–274]. Specifically, CAFs attract monocytes and promote their M2 polarization via the secretion of IL-8. This M2-like polarization can synergize with CAFs to restrain natural killer cells function [275]. CAFs also produce CCL2, CXCL12, IL-6, IL-10, glycoprotein CHI3L1, macrophage colony-stimulating factor to promote the migration of monocytes into tumor tissue and support their transdifferentiation into the M2 phenotype [276–282]. Interestingly, TAMs are reported to regulate the activation of CAFs by releasing CXCL12 and IL-6, thereby forming a positive loop to endorse cancer progression [282].

Analogous to the phenotypic macrophages, neutrophils can be roughly separated into two different polarized populations: N1 neutrophils with antitumor phenotype and N2 neutrophils with pro-tumor phenotype [283]. CAFs-derived IL-6 participated in the activation of STAT3 signal in tumor‑associated neutrophils (TANs), which sustained the survival and function of TANs and inhibited T cell’s attack ability via the PD1/PD-L1 signaling [284]. Cardiotrophin-like cytokine factor 1 derived from CAFs upregulated the CXCL6 and TGF-β expression levels in cancer cells, which promoted the polarization of the N2 neutrophil phenotype [285].

Myeloid-derived suppressor cells (MDSCs) are a heterogeneous cell population that consists of immature myeloid cells and myeloid progenitor cells, with immunosuppressive activity in tumor development. MDSCs can regulate both innate and adaptive immune responses [286, 287]. They are influenced by CAFs to inhibit the antitumor activity of effector T cell. In short, CAFs facilitate MDSCs generation and infiltration mainly by releasing multiple secretory factors including chemokine CCL2, CXCL1, CXCL2, CXCL12 and cytokine IL-6, TGF-β, etc. [288]

As an indispensable population of antigen-presenting cells, dendritic cells (DCs) are affected by CAFs to induce tumor cells immune evasion, usually accompanied by impaired DCs maturation and blocked antigen presentation [289]. CAFs are found to recruit DCs and confer them a capacity to produce indoleamine 2,3-dioxygenase (IDO). These DCs inhibit T cells proliferative ability and upregulate the production of Treg in a IL-6-STAT3-dependent manner [290]. Furthermore, CAFs assist the proliferation and migration of mast cells (MCs) by the CXCL12/CXCR4 axis and potentiate MCs protumorigenic function [291]. The CAFs precursors, stellate cells, can stimulate MCs to secrete IL-13 and tryptase, which creates a fibrotic TME and mediates restrained antitumor immunity [292]. Reciprocally, tryptase derived from MCs potentiates CAFs-induced early malignant morphology changes of prostate epithelial cells [293]. CAFs also induce natural killer (NK) cells dysfunction and mediate their functional and phenotypic alterations by releasing PGE2 and IDO [294, 295].

The implications of CAFs on adaptive immunity are mainly achieved by regulating T lymphocytes activity. The antigen crosspresentation driven by CAFs could negatively modulate T cells function and survival [296]. Mechanistically, PD-1 ligand 2 (PD-L2) expressed by CAFs induces T cell anergy and even death through the interaction with PD-1. CAFs also express FAS ligand (FASL) to induce the apoptosis of CD8^+^ T cell expressing FAS [297]. In addition, TGF-β was uncovered to abate the antitumoral immune via the exclusion of CD8^+^ T cells [298, 299]. CAFs-derived CXCL12 is also necessary for blocking the access of CD8^+^ T cell and the failure of the treatment of T-cell checkpoint antagonists [300, 301].

CAFs can directly interfere with T cell’s activity by regulating the expression of immune checkpoint molecules including PD-L1, PD-L2, B7-H3, and B7-H4. Among them, PD-L1 and PD-L2 are the best-studied types. They can bind to the PD-1 receptor on T cell surface to impair T cell’s function. [297, 302–304]. CAFs also affect Th cell subsets, mainly Th2 cell subpopulations, and Treg transformation to inhibit antitumor response [270].

### TA-MSCs and the immune system

MSCs are tightly correlated with both innate and adaptive immune, in particular mediating antitumor immune response. The immune modulatory functions of MSCs are mainly attribute to their capacity to block effector cells’ activated surface receptors expression, support regulatory cells expansion, and impair the maturation of antigen-presenting cells [305–308].

TA-MSCs isolated from cervical cancer dramatically repressed antigen-specific T cell recognition of tumor cells by cytotoxic T lymphocytes (CTLs) and provided immune protection for tumor cells growth. Mechanistically, TA-MSCs induced the downregulation of HLA class I molecules on cancer cells membrane in an IL-10-mediated manner, whereas HLA class I is important for the recognition by CTLs [309]. After co-culturing with MSCs, the proliferative potential of FoxP3^+^Treg was significantly enhanced, accompanied by the reduction of antitumor Th1 cytokines and the increase of Th2 cytokines, which mediated cancer cells immune evasion and contributed to disease progression. This finding can be partially explained by the elevated levels of MSC-generated TGF-β1 [310]. Another mechanism regarding MSCs restraining effective immune response is to destroy DCs mature as shown by the decreased expression of CD83 on DCs surface [311]. Furthermore, TA-MSCs usually exhibit a remarkable antiproliferative effect on mononuclear cells and abate NK cell activity [312].

Numerous chemokines participate in the communication between TA-MSCs, tumor cells and macrophages, which is driven by the HIF signal and substantially stimulate the invasion and metastasis of MDA-MB-231 cell [313]. On the one hand, CXCL10 secreted by TA-MSCs bind to its cognate receptors CXCR3 presented in cancer cells, and simultaneously, CXCL16 derived from cancer cell bind to CXCR6 on TA-MSCs surface, eventually potentiating the recruitment of TA-MSCs into tumor areas. On the other hand, TA-MSCs release CCL5 to bind to CCR5 on breast cancer cells, and then, signal-received cancer cells express CXCL12 to drive the migration and recruitment of TAMs and MDSCs [313]. Interestingly, there are bidirectional interactions between TA-MSCs and immune cells. TA-MSCs are plastic and can be modified by CD4^+^ T cell to induce tumor growth [314]. Following stimulating by CD4^+^ T cell, the immunophenotype of TA-MSCs undergoes significant changes, as they acquire the ability to overexpress PD-L1 in a STAT3-dependent manner and subsequently activate cancer cell-intrinsic PD-1/mTOR signaling to assist gastric cancer development [314].

### Pericytes and the immune system

Pericytes exert their immunosuppressive functions by releasing multiple factors such as nitric oxide, IL-6, IL-33, CXCL12, PGE2 and TGFβ [218, 315]. Furthermore, the accumulation of pericytes affects cytotoxic lymphocytes activity, as they hinder allogeneic and mitogen-activated T cell responses in vitro[316]. Meanwhile, Bose et al. firstly confirmed that tumor-derived pericytes had a negative influence on the proliferation and activation of CD4^+^ T cell as well as resulted in CD4^+^ T cell dysfunction even anergy in response to antigen in an IL-6-dependent manner, which possibly hampered effective antitumor immune responses and shielded tumor cells from the host immune attack [317]. Pericytes are also responsible for recruiting MDSCs into the stroma to create an immunosuppressive surrounding that is favorable for tumor growth [318].

## Targeted therapy based on tumor stroma

Traditionally, the rationale for anticancer therapies mainly focuses on eliminating tumor cells only while largely ignoring the ambient non-malignant-cell components of a tumor. In recent years, we have witnessed a great upgrade of precision medicine, and among all, molecular targeted therapy has been widely developed and introduced into clinical practice. Also, cancer initiation, progression and metastasis usually elicit a broad spectrum of dynamic evolutions and alterations in host tissues, which contributes to establishing complicated stromal surroundings that in turn cover a wide range of tumor cell activities and support cancer development. Accordingly, tumor stroma may be a fertile ground for developing effective therapeutic strategies to hopefully augment existing treatment options and realize personalized cancer therapy, especially for those stromal-rich and refractory cancers.

**Fig. 6:**
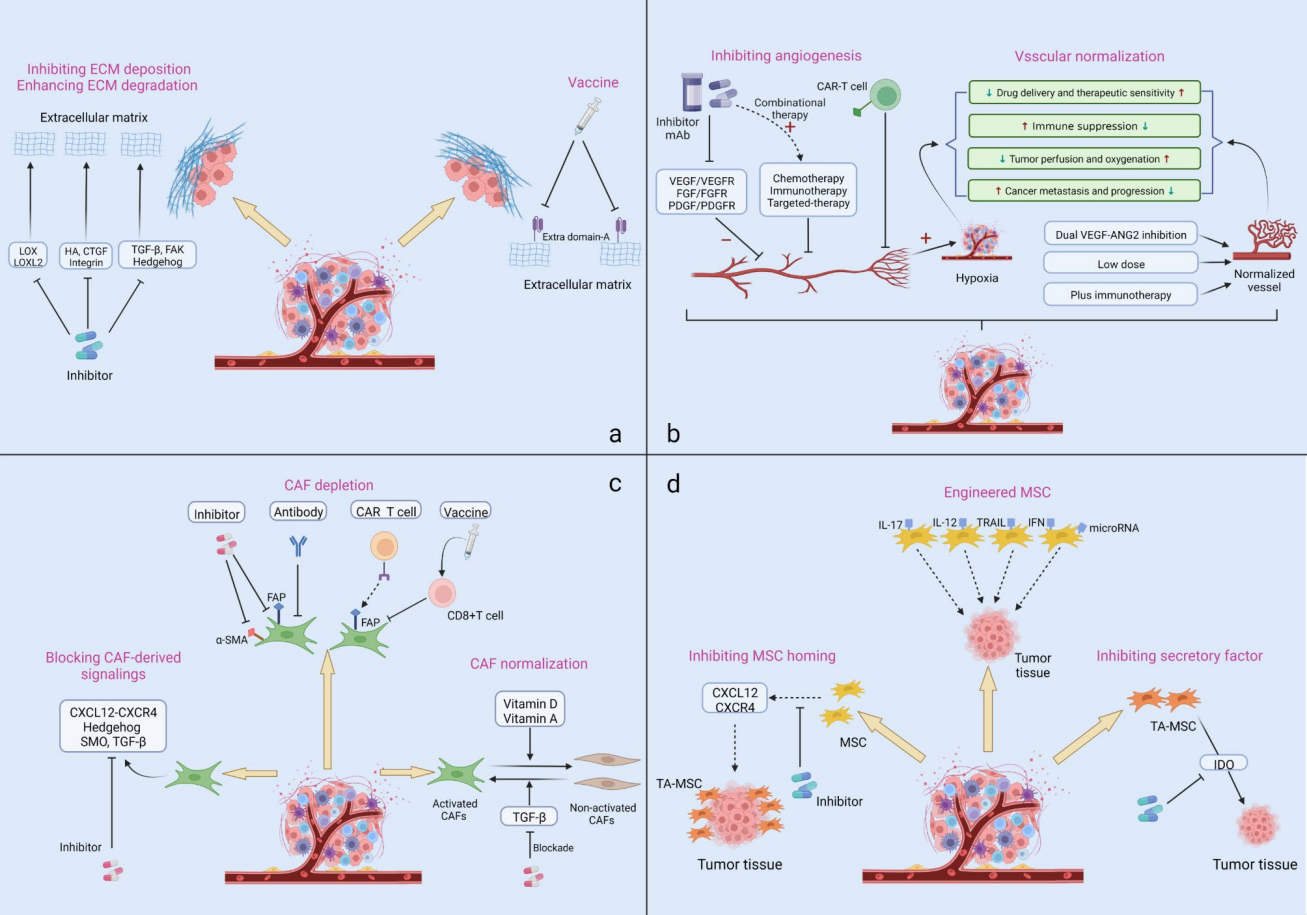
Therapeutic approaches based on the stromal components

Approaches to targeting the tumor stroma include directly targeting both cellular or noncellular elements located in the stroma, disrupting and inhibiting related secretory factors and signaling pathways, and recently proposed reshaping or normalizing the tumor stroma that aims to slow or reverse tumor progression (Fig. 6). Herein, we summarize recent advancements targeting stromal components and highlight related potential therapeutic values, with the aim to promote the leap from bench to bedside.

### Targeting the ECM

Compared with the normal ECM, tumor ECM is more abundant, denser and stiffer. The tumor ECM typically undergoes a series of changes such as deposition, degradation, and post-translational modification [319]. To date, several strategies have been designed to inhibit or decrease the ECM with tumor-promoting functions, such as inhibiting the ECM synthesis and deposition, enhancing the degradation of different ECM components, and blocking signaling molecules that contribute toward cell-matrix interactions and protumorigenic feedback. Some targeted drugs are being assessed in clinical trials (Table 1).

**Table 1:** Clinical trials targeting the ECM

Target	Drug	Combination	Condition	Phase	Status	Trial number
LOXL2	Simtuzumab	Gemcitabine	Pancreatic Cancer	II	Completed	NCT01472198
		FOLFIRI	Colorectal Cancer	II	Terminated	NCT01479465
	PAT-1251	No	Healthy	I	Completed	NCT02852551
LOX	PXS-5382A	No	Healthy	I	Completed	NCT04183517
Hyaluronic acid	PEGPH20	Pembrolizumab	Pancreatic Cancer/Pancreatic Ductal Adenocarcinoma	II	Recruiting	NCT03634332
		Gemcitabine	Pancreatic Adenocarcinoma Non-resectable	II	Terminated	NCT02910882
		Pembrolizumab	NSCLC/Gastric Cancer	I	Completed	NCT02563548
		Cetuximab	Pancreatic Cancer	Not Applicable	Completed	NCT02241187
		Avelumab	Pancreatic Ductal Adenocarcinoma/Pancreatic Cancer	I	Terminated	NCT03481920
		Docetaxel	Non-small Cell Lung Cancer	I	Terminated	NCT02346370
		Eribulin mesylate	Metastatic Breast Cancer	I/II	Terminated	NCT02753595
		No	Solid Tumor	I	Completed	NCT01170897
		Gemcitabine	Pancreatic Cancer	I/II	Completed	NCT01453153
		FOLFIRINOX	Metastatic Pancreatic Adenocarcinoma	I/II	not recruiting	NCT01959139
		No	Solid Tumor	I	Completed	NCT00834704
		CIS/GEM/Atezolizumab	Cholangiocarcinoma	I	Terminated	NCT03267940
		Nabpaclitaxel/Gemcitabine	Metastatic Pancreatic Cancer	II	Completed	NCT01839487
		Atezolizumab	Pancreatic Ductal Adenocarcinoma	II	Terminated	NCT03979066
		Gemcitabine/Nab-paclitaxel	Adenocarcinoma	II	Terminated	NCT02487277
		NabPaclitaxel/Gemcitabine	Pancreatic Ductal Carcinoma	III	Terminated	NCT02715804
CTGF	Pamrevlumab	Gemcitabine combined with nab-paclitaxel	Metastatic Pancreatic Cancer	III	Recruiting	NCT04229004
	Pamrevlumab	Pamrevlumab,Gemcitabine, Nab-paclitaxel, or Pamrevlumab, FOLFIRINOX	Pancreatic Cancer Non-resectable	III	Active, not recruiting	NCT03941093
Integrin	Cilengitide	Temozolomide,radiotherapy	Glioblastoma	III		NCT00689221
	ATN-161	Carboplatin	Malignant glioma	I/II	Completed	NCT04177108
	MEDI-522	Dacarbazine	Metastatic melanoma	II	Completed	NCT00066196
TGF-β	Fresolimumab	Radiation Therapy	Metastatic Breast Cancer	II	Completed	NCT01401062
		Radiation Therapy	Non-Small Cell Lung Carcinoma	I/II	Completed	NCT02581787
FAK	Defactinib/PF-04554878	No	Malignant Pleural Mesothelioma	II	Terminated	NCT01870609
		No	Non-Small Cell Lung Cancer	II	Completed	NCT01951690
		No	Solid Tumor	I	Completed	NCT00787033
		No	Non-Hematologic Malignancies	I	Completed	NCT01943292
		No	Advanced tumor	II	Active, not recruiting	NCT04439331
		VS-6766	Ovarian Cancer	II	Recruiting	NCT04625270
		Paclitaxel	Ovarian Cancer	I	Completed	NCT01778803
		VS-6766	Lung cancer/ ovarian cancer/ endometrioid carcinoma / pancreatic cancer	I	Recruiting	NCT03875820
		VS-6766	Non-Small Cell Lung Cancer	II	Recruiting	NCT04620330
		Pembrolizumab, Gemcitabine	Solid tumors / pancreatic cancer	I	Completed	NCT02546531
		Paclitaxel, carboplatin	Ovarian cancer	I/II	Recruiting	NCT03287271
Hedgehog	Sonidegib (LDE225)	Docetaxel	Triple Negative (TN) Advanced Breast Cance	I	Completed	NCT02027376
		No	Basal Cell Carcinoma	II	Completed	NCT01327053
		Gemcitabine	Pancreatic Cancer	I	Completed	NCT01487785
		No	Hepatocellular Carcinoma	I	Completed	NCT02151864
		Etoposide or Cisplatin	Small Cell Lung Cancer	I	Completed	NCT01579929
		Pembrolizumab	Advanced Solid Tumors	I	Recruiting	NCT04007744
		Fluorouracil, Leucovorin, Oxaliplatin, Irinotecan	Pancreatic Cancer	I	Completed	NCT01485744
	Vismodegib	No	Basal Cell Carcinoma	II	Completed	NCT03035188
		Modified FOLFOX or FOLFIRI	Metastatic Colorectal Cancer	II	Completed	NCT00636610
		Gemcitabine Hydrochloride	Pancreatic Cancer	II	Completed	NCT01195415
		Gemcitabine or nab-Paclitaxel	Pancreatic Cancer	II	Completed	NCT01088815

One of the promising options for inhibiting ECM deposition is to disrupt its crosslinking and stabilization. Among these strategies, targeting lysyl oxidase (LOX) activity that is frequently upregulated in diverse cancer types and responsible for catalyzing collagen crosslinking is emerging as a optimal one, which can reduce the stroma density and consequently enhance the outcome of anticancer treatment [320–326]. Simtuzumab is an antibody targeting LOXL2 and has been tested clinically to appraise its efficacy and safety. A phase II trial of simtuzumab combined with gemcitabine was conducted to treat adult patients with metastatic pancreatic adenocarcinoma. Although this therapeutic regimen was tolerable, the progression-free survival (PFS), overall survival (OS) or objective response rate(ORR) in patients have not been improved [327] (NCT01472198). Simtuzumab in combination with FOLFIRI was also used to treat patients with colorectal cancer, and the ultimate result suggested that addition of simtuzumab did not improve the clinical outcome [328] (NCT01479465). PAT-1251 and PXS-5382 A are developed to target LOXL2 and LOX respectively, and clinical trials have studied their safety and tolerability in healthy adult subjects. Their anticancer potencies need to be rigorously explored (NCT02852551, NCT04183517).

Another rational approach to targeting ECM deposition or degradation is to degrade hyaluronic acid (HA) that typically accumulates cancer and can mechanically increase the ECM elastoviscosity [329–332]. PEGPH20 was designed to inhibit HA and underwent clinical trials as a single agent or in combination with other therapeutic drugs. Two similar studies have been conducted to evaluate its safety, tolerability and pharmacokinetics in patients with solid tumor, but the results have not been disclosed (NCT01170897, NCT00834704). A phase Ib study reported the effect of docetaxel in combination with PEGPH20 in patients with lung cancer. This strategy seemed to manifest an acceptable safety profile [333] (NCT02346370). In a randomized phase II trial, researchers investigated the effects of PEGPH20 in combination with standard nab-paclitaxel plus gemcitabine (PAG) to treat pancreatic cancer patients. The results showed that patients with HA-high tumors who received PAG had the largest FAS improvement, and importantly, the related clinical data also supported the potential application of tumor HA as a predictive biomarker for cancer patients [334] (NCT01839487). Notwithstanding, the results have been mixed. Owing to the negative trial outcome that didn’t meet its primary end point of OS, a similarly subsequent phase III study had to be terminated [335] (NCT02715804). Another phase IB/II randomized study tested the clinical efficacy of PEGPH20 with modified fluorouracil, leucovorin, irinotecan, and oxaliplatin (mFOLFIRINOX) to treat patients with metastatic pancreatic cancer. Unfortunately, compared with mFOLFIRINOX alone, this therapeutic scheme led to increased toxicity and decreased treatment duration, suggesting that the addition of PEGPH20 yielded detrimental effect in patients unselected for tumor HA status [336] (NCT01959139).

The connective tissue growth factor (CTGF) is responsible for enhancing matrix deposition in cancers, and anti-CTGF therapy can reduce matrix deposition in murine pancreatic cancer model [337]. To date, using pamrevlumab to target CTGF in patients with pancreatic cancer have entered phase III clinical trials (NCT03941093, NCT04229004). Integrin is a critical mechanosignal transducer that can perceive the ECM mechanical force and mediate signal transductions to intracellular proteins. Hence, targeting integrin may be a promising approach to delaying tumor progression, and meanwhile, a series of clinical trials have been launched continually to evaluate its therapeutic prospects (NCT00689221, NCT04177108, NCT00066196).

Among all signaling molecules that are involved in ECM deposition, TGF-β represent an optimal target to inhibit collagen synthesis and subsequently ECM deposition. Several TGF-β-targeted drugs have been actively assessed in clinic to potentiate antitumor effects [338, 339] (NCT01401062, NCT02581787). An alternative method is to target FAK, an important downstream effector of integrins [340]. FAK inhibitors have shown antitumor activity in preclinical studies [341–343]. Based on the above successful practices, defactinib (also known as PF-04554878) has been tested in phase clinical trials, mainly in malignant pleural mesothelioma and advanced solid tumors. Even though this drug was well tolerated, using defactinib alone or in combination with other therapies to treat patients with different cancers showed limited outcome or even failed to show clinical benefits [344–347] (NCT01951690, NCT00787033, NCT01870609). Furthermore, a previous study uncovered that blocking the fibrotic Hedgehog signaling pathway could decrease fibrosis in cancer, which enhanced the delivery of chemotherapy and contributed to prolonged survival times in tumor-bearing mice [56]. Hitherto, several Hedgehog inhibitors such as vismodegib and sonidegib (LDE225) have been studied in clinical trials to mainly treat patients with basal cell carcinoma and solid tumors, which are summarized in Table 1.

Cancer vaccine is emerging as a promising therapeutic strategy for solid tumors and being intensively evaluated in both preclinical and clinical studies [348]. Several ECM components have recently been used as antigens for designing cancer vaccine. During tumor matrix remodeling, the alternatively spliced extra domain-A (ED-A) of fibronectin was reported to reexpress, which enabled them to become an ideal target. Targeting ED-A with immunization in the therapeutic condition could inhibit cancer metastasis and decrease the tumor burden, which suggested that the ECM might behave as a suitable candidate for designing effective cancer vaccines and warranted further study in clinical trials [349].

### Targeting cancer vasculature

In 2004, the American FDA granted an unprecedented approval to a humanized anti-VEGFA monoclonal antibody, named as bevacizumab, to treat patients with metastatic colorectal cancer [350]. Since then, targeting cancer vessels has aroused great interest of an increasing number of scientists and been utilized in clinical practices. The conventional tactic is to inhibit proangiogenic signaling or factors activity, but in some conditions, this application has not yielded long-term clinically survival benefits and even unexpectedly promotes drug resistance or limits agent delivery, ultimately leading to tumor metastasis [351, 352]. As such, an attractive possibility is remodeling aberrant tumor blood vessels, which can restore the structure and function of vasculature and then improve the drug penetration, as well as achieve better outcomes, currently known as “vascular normalization” [351, 353]. In this section, we summarize the clinical trial progress in antiangiogenic therapies and the strategies for vascular normalization (Table 2).

**Table 2:** Some major clinical trials targeting cancer vasculature

Target	Drug	Combination	Condition	Phase	Status	Trial number
VEGF/VEGFR	Bevacizumab	No	Solid tumors	IV	Completed	NCT01588184
	Bevacizumab	Chemotherapies	Ovarian cancer	III	Active, not recruiting	NCT00565851
	Bevacizumab	Erlotinib	Lung cancer	II	Completed	NCT01562028
	Bevacizumab	Erlotinib	Hepatocellular Carcinoma	II	Completed	NCT01180959
	Bevacizumab	Niraparib	Ovarian Cancer	I/II	Completed	NCT02354131
	Olaparib	Chemotherapies	Ovarian Cancer	III	Active, not recruiting	NCT02477644
	Olaparib	Enzalutamide, abiraterone acetate	Prostate Cancer	III	Active, not recruiting	NCT02987543
	Bevacizumab	Osimertinib	Lung Cancer	I/II	Completed	NCT02803203
	Bevacizumab	Osimertinib	Lung Cancer	III	Recruiting	NCT04181060
	Ramucirumab	Erlotinib, Gefitinib, Osimertinib	Metastatic NSCLC	III	Active, not recruiting	NCT02411448
	Ramucirumab	Paclitaxel	Gastric Adenocarcinoma	III	Completed	NCT01170663
	Ramucirumab	No	Gastric Cancer and Adenocarcinoma	III	Completed	NCT00917384
	Ramucirumab	No	Hepatocellular Carcinoma	III	Completed	NCT01140347
	Ramucirumab	No	Hepatocellular Carcinoma	III	Completed	NCT02435433
	Ramucirumab	No	Hepatocellular Carcinoma	III	Completed	NCT02435433
	Aflibercept	FOLFIRI	Metastatic Colorectal Cancer	III	Completed	NCT00561470
	Aflibercept	Levofolinate, Irinotecan, 5-FU	Metastatic Colorectal Cancer	II	Completed	NCT01882868
	Aflibercept	Capecitabine	Metastatic Colorectal Cancer	I/II	Completed	NCT01661972
TKI	Sorafenib	No	Hepatocellular Carcinoma	III	Completed	NCT00692770
	Sunitinib	AGS-003	Kidney Cancer	II	Completed	NCT00678119
	Sunitinib	Nivolumab, Pazopanib, Ipilimumab	Renal Cell Carcinoma	I	Completed	NCT01472081
	Pazopanib	No	Ovarian Cancer	I/II	Completed	NCT01238770
	Pazopanib	No	Renal Cell Carcinoma	IV	Completed	NCT01521715
	Pazopanib	Paclitaxel	Ovarian Cancer	II	Completed	NCT01644825
	Pazopanib	GSK1120212	Solid Tumors, Thyroid Cancer	I	Completed	NCT01438554
ANG-VEGF	Vanucizumab	Atezolizumab	Solid Tumors	I	Completed	NCT01688206
	Vanucizumab	Bevacizumab, Selicrelumab	Solid Tumors	I	Completed	NCT02665416
Plus immunotherapy	Bevacizumab	Nivolumab, Rucaparib	Peritoneal Cancer,Ovarian Cancer, Fallopian Tube Cancer	II	Recruiting	NCT02873962
	Bevacizumab	Nivolumab and chemotherapies	Non-small Cell Lung Cancer	I	Completed	NCT01454102
	Bevacizumab	Pembrolizumab	Clear Cell Renal Carcinoma	I/II	Completed	NCT02348008
	Bevacizumab	Pembrolizumab	Glioblastoma	II	Completed	NCT02337491
	Bevacizumab	Pembrolizumab	Ovarian, Fallopian Tube, or Primary Peritoneal Cancer	II	Completed	NCT02853318
	Bevacizumab	Durvalumab	Glioblastoma	II	Completed	NCT02336165
	Bevacizumab	Durvalumab	Metastatic Breast Cancer	I	Completed	NCT02802098.
	Bevacizumab	Tremelimumab	Colorectal Cancer With Liver Metastases	I	Active, not recruiting	NCT02754856
	Vanucizumab	Atezolizumab	Metastatic Solid Tumors	I	Completed	NCT01688206

Among all proangiogenic signalings, VEGF/VEGFR is the best-studied pathway, and related mAb or inhibitors have been widely used in clinic. Bevacizumab that can target VEGF-A and inhibit its interaction with VEGFR-1 and − 2 has been tested in various human cancer types, both as monotherapy and in combination with other antitumor drugs [354, 355]. A recent clinical study evaluated the safety of long-term administration of bevacizumab in patients with solid tumors. No treatment-related adverse effect was happened and patients obtained clinical benefit over an extended period (NCT01588184) [356]. However, previous studies indicated that side effects usually increased when bevacizumab was combined with chemotherapies [357, 358]. These opposite results indicate that the clinical responses and toxic side effects may depend on the specific therapeutic schemes and conditions [359]. As for its therapeutic outcomes in combination with chemotherapies, some published meta-analyses assessed the additional effect of chemotherapy plus bevacizumab, and the results indicated that, compared to chemotherapy alone, the combinational strategy improved the PFS and OS in cancer patients [360–363]. Nonetheless, disappointing outcomes are still existed. In a large randomized phase III trial, researchers assessed the effect of standard chemotherapy with or without bevacizumab for women with newly diagnosed ovarian cancer, and the antitumor response was not as promising as initially hoped with no increased OS in the study population was observed [364]. This contradictory phenomenon may be attributed to the different dose usage and particular cancer types [365].

Aside from in combination with chemotherapy, bevacizumab plus targeted therapy often exhibits antitumor activity and yields clinical benefits in cancer patients. For example, erlotinib, an epidermal growth factor receptor (EGFR) tyrosine kinase inhibitor, has shown synergistic effects when combined with anti-VEGF therapies, mainly in patients with advanced non-small cell lung cancer and colorectal cancer [366–370]. Compared to erlotinib alone in EGFR-positive NSCLC patients, the combinational utilization of bevacizumab plus erlotinib brings clinical benefits to patients with the improvement of their progression-free survival (NCT02759614, NCT01562028) [371–373]. Furthermore, this combined regimen has also been verified in liver cancer patients and has shown a signal of survival benefit, which supports the further clinical studies of this strategy (NCT01180959) [374]. In addition to erlotinib, many other molecular targeted drugs such as olaparib, niraparib and osimertinib in combination with bevacizumab have been approved and introduced across several indications in the clinic, and it was feasible to expand the application of these dual-targeted therapies owing to the observation of progression-free survival benefits in patients (NCT02477644, NCT02354131, NCT02987543, NCT02803203 NCT04181060) [375–378].

Other anti-VEGF signaling drugs have also been assessed in the clinic, both as single agents and in combination with chemotherapy or targeted therapy. Ramucirumab, also known as Cyramza, is a humanized antibody approved by the FDA that targets the VEGFR-2 extracellular domain, and exhibits some degree of efficiency in prolonging PFS and OS in patients with lung cancer, gastro-oesophageal junction adenocarcinoma, and liver cancer (NCT02411448, NCT01170663, NCT01140347) [379–382]. Aflibercept is a recombinant fusion protein that can inhibit the combination of VEGF and VEGFR, and has been approved in combination with FOLFIRI to treat patients with metastatic colorectal cancer (NCT00561470, NCT01882868, NCT01661972) [383–386].

Alternative options that may represent attractive therapies are using tyrosine kinase inhibitors (TKIs) and targeting other proangiogenic signalings, such as inhibiting FGF/FGFR and PDGF/PDGFR, which have been tested or are undergoing phase clinical trials [387]. Among those approved TKIs, sorafenib has gradually become the research hot spot for the treatment or alleviation of a variety of cancer conditions, especially in liver cancer [388, 389]. Other TKIs such as sunitinib and pazopanib, together with inhibitors of FGF/FGFR and PDGF/PDGFR axis have also generated some degree of clinical benefits [387], as listed in Table 2.

In spite of some promising results have been observed, the clinical activity of anti-angiogenic therapies is usually partial and eventually, followed by relapse. Mechanistically, previous methods that inhibit angiogenesis are cutting off their blood supply, but this strategy simultaneously exacerbates the formation of an anoxic TME, leads to the increased compensatory proangiogenic factors production, and creates an immunosuppressive circumstances, thus facilitating pathological angiogenesis and disease progression [390–393]. Therefore, “vascular normalization” that aims to judiciously use antiangiogenesis treatment rather entirely destruction or excessive pruning and keep the balance between proangiogenic and antiangiogenic signalings becomes an advisable direction and is hopefully to accelerate the development of antitumor therapy due to its potential to improve tumor oxygenation and perfusion, enhance the efficiency of drug delivery and delay tumor progression [392].

Long-term vascular-targeted therapies in high doses usually give rise to tumor hypoxia, and meanwhile, some specific cancer types are susceptible to anti-VEGF treatments, which underscores the importance of selecting appropriate doses and time ranges of anti-angiogenic drugs’ administration to achieve vascular normalization as initially expected, termed as “normalization window” [394, 395]. High-dose administration of anti-VEGF agents cannot lead to beneficial outcomes, and instead forms an immunosuppressive TME accompanied by the recruitment of nonclassical Ly6C^low^ monocytes, which also promotes the occurrence of therapeutic resistance to anti-VEGF [396–398]. Clinically, patients with rectal cancer receiving a relatively low dose of bevacizumab (5 mg/kg) had favorable delivery efficiencies and enhanced pericyte coverage of blood vessels, whereas patients receiving higher dosage of bevacizumab (10 mg/kg) did not exhibit the same benefits, suggesting that the choice of drug dosage is a key consideration in cancer treatment [399]. Likewise, low-dose intensity of bevacizumab confers a greater survival benefit than the usage of high-dose and can be regarded as a significant independent prognostic survival factor in glioblastoma patients [400–402]. In the context of the “normalization window”, antiangiogenesis therapies have synergistic and reinforced effects with other anti-cancer therapeutic modalities including immunotherapy and chemotherapy [403]. However, it has been recognized that the dosage of anti-VEGF targeted drugs that can achieve the window is relatively narrow and their effects often vary among different cancer types [232, 404]. Hence, how to best utilize the anti-angiogenic strategy to benefit patients with cancer is the chief question we need to overcome and merits further mechanistic and functional investigation.

In addition to legitimately identifying the dosage of anti-angiogenic drugs, another noteworthy question is that the process of vascular normalization is transient and reversible, with lasting for several days or months after therapy began in diverse cancer types. In this regard, researchers unexpectedly found a compensatory mechanism in which ectopic expression of angiopoietin (Ang)-2 could inhibit vessel normalization to diminish the beneficial effects of VEGF-VEGFR signaling blockade [405]. Dual inhibition of VEGF-ANG2 has been explored and exhibited prolonged normalization window in mouse model of glioblastoma. Dual VEGF-ANG2 blockade also contributed to antitumor immunity [406–408]. This finding motivated the initiation of multiple clinical trials using dual anti-VEGF and ANG2 inhibitors for tumors, with some trials have shown clinically survival benefits.

Immunotherapy is gradually becoming a central focus of cancer therapy and represents a suitable method in advanced solid tumors. Interestingly, tumoral vascular normalization has the potential to improve the infiltration of diverse immune effector cells, and vice versa as the discovery that the functional stimulation of immune cells can normalize tumor vessels, which establishes a bidirectionally positive feedback loop for antitumor effects and provides a novel combined option for antitumor treatment [409–412]. A phase II trial assessed the effect of nivolumab (anti-PD-1 mAb) in combination with bevacizumab in patients with different cancer types and showed clinical benefit with improved ORR or durable response (NCT02873962, NCT01454102) [413, 414]. Some others anti-PD-1 mAb, such as pembrolizumab and durvalumab in combination with bevacizumab, have also been tested in clinical trials, as summarized in Table 2. In addition to anti-PD-1 drugs, anti-CTLA-4-mAb plus vascular-targeted agents are also utilized to treat patients with cancers, but the related results have not been disclosed yet (NCT02754856, NCT01688206). Noteworthy, based on the advances and successful practices of engineered chimeric antigen receptor (CAR) T cells therapy, different CAR designs have been exploited to fight against various diseases, in particular malignancies. Therefore, using CAR-T cells to target multiple antigens on tumor vasculature may provide new opportunities for the development of anti-angiogenic therapy [415].

Taken together, since the first angiogenesis inhibitor bevacizumab was approved for cancer treatment, numerous vascular-targeted drugs have been designed and exploited in clinic. However, anti-VEGF or targeting other proangiogenic signalings as monotherapy sometimes yields limited clinical outcomes or even results in metastasis, and therefore caution must be taken. Furthermore, realizing vascular normalization without excessive pruning opened new avenues for cancer therapy. One of these approaches is to choose a rational dosage and time range of anti-angiogenic drugs. Further prospective and randomized trials using lower dose of vascular-targeted drugs are warranted. It is also worth noting that judicious use of immune checkpoint blockade together with angiogenesis inhibitors has potential to improve cancer treatment. The next goal of both preclinical and clinical studies is finding the most reasonable combinations to exert more robust anti-tumor immune responses and reduce toxic side effects. The possibilities of this field are virtually endless.

### Targeting CAFs for cancer therapy

Numerous previous researches identified various mechanisms of CAFs tumor-promoting functions. Clinically, it was confirmed that the infiltration of activated CAFs was closely related to worse prognosis, resistance to multiple therapies, and even disease recurrence in cancer patients [416–420]. Hence, targeting CAFs has evolved as one of the appealing strategies for cancer intervention and is expected to provide oncologists with clinical decision-making (Table 3).

**Table 3:** Clinical trials targeting the CAFs

Target	Drug	Combination	Condition	Phase	Status	Trial number
FAP-expressing cells	Simlukafusp alfa (RO6874281)	Atezolizumab and chemotherapy	Advanced and/or Metastatic Solid Tumors	II	Completed	NCT03386721
	Simlukafusp alfa (RO6874282)	Trastuzumab and Cetuximab	Solid tumor	I	Active, not recruiting	NCT02627274
	Sibrotuzumab (BIBH 1)	No	Metastatic Colorectal Cancer	II	Completed	NCT02198274
	FAP-specific CAR-T	No	FAP-Positive Malignant Pleural Mesothelioma	I	Completed	NCT01722149
		No	Malignant Solid Tumors	I	Recruiting	NCT03932565
Vitamin D receptor	Calcipotriol	5-fluorouracil	Skin cancer precursor immunotherapy	I	Completed	NCT02019355
	Paricalcitol	Docetaxel, ixabepilone, and paclitaxel	Metastatic Breast Cancer	I	Completed	NCT00637897
	Paricalcitol	Gemcitabine, Nab-paclitaxel	Metastatic Pancreatic Cancer	I/II	Recruiting	NCT03520790
Vitamin A metabolism	ATRA	Gemcitabine and Nab-paclitaxel	Pancreatic Adenocarcinoma	I	Completed	NCT03307148
		No	Advanced Adenoid Cystic Carcinoma	II	Completed	NCT03999684
		Paclitaxel and Cisplatin	Non-small Cell Lung Cancer	III	Unknown	NCT01041833
		Interferon-Alpha 2a	Recurrent Neuroblastoma or Wilms’ Tumor	II	Completed	NCT00001509
TGFβ	LY2157299(galunisertib)	Nivolumab	Solid Tumor; Non-Small Cell Lung Cancer; Hepatocellular Carcinoma Recurrent	I/II	Completed	NCT02423343
		Gemcitabine	Advanced or Metastatic Unresectable Pancreatic Cancer	I/II	Completed	NCT01373164
		Sorafenib, Ramucirumab	Hepatocellular Carcinoma	II	Completed	NCT01246986
		Durvalumab	Metastatic Pancreatic Cancer	I	Completed	NCT02734160
		Radiation, Temozolomide	Malignant Glioma	I/II	Completed	NCT01220271
		Capecitabine, Fluorouracil	Rectal Cancer	II	Active, not recruiting	NCT02688712
		Lomustine	Glioblastoma	II	Active, not recruiting	NCT01582269
	Minnelide	No	Advanced Gastrointestinal Tumors	I	Completed	NCT01927965
		No	Pancreatic Cancer	II	Completed	NCT03117920
	AP 12,009	No	Pancreatic Neoplasms, Melanoma, Colorectal Neoplasms	I	Completed	NCT00844064
CXCR4	Motixafortide (BL-8040)	Pembrolizumab, chemotherapy	Metastatic Pancreatic Cancer	II	Active, not recruiting	NCT02826486
	AMD3100 (plerixafor)	No	Advanced Pancreatic, Ovarian and Colorectal Cancers	I	Completed	NCT02179970
		Mozobil	Children Cancer, Solid Tumor	II	Completed	NCT01225419
Hedgehog	Itraconazole	No	Basal Cell Carcinoma (BCC), Skin Cancer	II	Completed	NCT01108094
	Vismodegib	No	Basal Cell Carcinoma	II	Completed	NCT01700049
		Gemcitabine hydrochloride	Pancreatic Cancer	I/II	Completed	NCT01064622
		Gemcitabine Hydrochloride	Pancreatic Cancer	II	Completed	NCT01195415
		Cisplatin, Cixutumumab, Etoposide	Lung cancer	II	Completed	NCT00887159
	LDE225 (sonidegib)	Etoposide and Cisplatin	Lung cancer	I	Completed	NCT01579929
		Paclitaxel	Solid Tumor	I	Completed	NCT01954355
		Docetaxel	Breast cancer	I	Completed	NCT02027376

At present, one strategy that has already been tested is CAFs-depletion by targeting their cell surface markers. Fibroblast activation protein-α (FAP), an integral serine protease specifically expressed by CAFs, participates in nearly all steps of the carcinogenic process [421–423]. High-level expression of FAP can predict poor prognosis in high-grade serous ovarian cancer [424]. The depletion of FAP^+^ cells inhibits tumor growth primarily achieved by augmenting anti-tumor immunity [425, 426]. Meanwhile, widespread efforts are underway to realize the translation of this plausible approach into practice. It has been established that a DNA vaccine exclusively targeting FAP could suppress primary and metastatic tumor growth, promote the uptake of chemotherapeutic drugs and prolong the survival of tumor-bearing mice primarily by inducing CD8^+^ T cell-mediated killing of CAFs [427, 428]. The combinational use of FAP-DNA vaccine and other tumor antigen-specific DNA vaccines showed synergic effects of anti-tumor immunity characterized by increased CD8^+^ T cell infiltration and decreased macrophage infiltration [429]. Additionally, FAP-CAR-T cell therapies have been engineered to treat solid tumors in preclinical studies [430]. In the mouse model, adoptively transferred FAP-specific CAR-T cells inhibited FAP^+^ CAFs activity and delayed the proliferation and growth of multiple types of subcutaneously transplanted tumors without the observation of distinct toxic signs [431]. A phase I trial was conducted to evaluate the safety of a fixed single dose of 1 × 10^6^ adoptively transferred FAP-specific CAR-T cells given directly in the pleural effusion (NCT01722149), but the result was still unavailable [432]. A bispecific FAP-CD40 antibody that could induce CD40 stimulation solely in the presence of FAP was designed, which induced predominantly intratumoral immune activation and exhibited well tolerance [433]. Furthermore, FAP-targeted in combination with near-infrared photoimmunotherapy were shown to recover the sensitivity to chemotherapy in CAF-rich tumors and induce tumor regression [434]. Some others FAP-targeted drugs or inhibitors such as RO6874281, PT630 and UAMC-1110 have been verified in several preclinical studies [423, 435, 436], and currently some of these agents have been advanced to testing in clinical trials (NCT03386721, NCT02627274 and NCT02198274).

The high expression of α-smooth muscle actin (α-SMA) is another prominent characteristic of CAFs, and its expression level has been identified as a novel biomarker of resistance to trastuzumab early-stage of HER2-positive breast cancer [437]. Specific targeting α-SMA with docetaxel-conjugate nanoparticles increased drug delivery efficiency by enhancing vascular perfusion and reduced cancer metastases [438]. In the myofibroblast-depleted mouse PDAC model, selective depletion of the α-SMA^+^ fibroblasts suppressed angiogenesis but led to enhanced tumor hypoxia and induced cancer stem cell-like phenotype. This selective depletion also contributed toward CD3^+^Foxp3^+^Treg cells infiltration into the tumor stroma, which ultimately increased tumor aggressiveness and reduced animal survival [20]. These contradictory findings emphasize the importance of targeting stromal cells with caution, and suitable targeting α-SMA rather than complete depletion may deserve further exploration.

Instead of direct CAFs depletion through their cell surface markers, the normalization of activated CAFs that aims to reprogram pro-tumorigenic CAFs into a non-activated or quiescent state represents a plausible option. It has been shown that using vitamin D receptor (VDR) ligand calcipotriol or all-transretinoic acid (ATRA) could achieve CAFs normalization. Specifically, VDR servers as a master genomic suppressor of pancreatic stellate cells (PSC) activation state and has the potential to revert PSCs into quiescent state. In this context, calcipotriol destroyed several tumor-supporting signaling pathways and enhanced the effects of chemotherapy in multiple mouse tumor models, which hindered the tumor-stroma interplay and tumor proliferation [439–442]. Some vitamin D analogues (e.g. calcipotriol and paricalcitol) are being or have been tested in the clinic (NCT02019355 [443], NCT00637897, NCT03520790). Moreover, ATRA is an active metabolite of vitamin A and is intensively studied because of its ability to restore the mechanical quiescence of PSCs and to inhibit aggressive tumors progression. The use of ATRA can suppress force-mediated extracellular matrix remodeling, block pro-tumorigenic signaling pathways and assist the migration of CD8^+^ T cells to tumor sites and juxtatumoral stromal compartments [300, 444–446]. Currently, ATRA has been evaluated in several clinical trials to treat patients with solid cancers, whether in combination or not with chemotherapies (NCT03307148, NCT03999684, NCT01041833) [447–450].

TGF-β also plays crucial roles in CAFs activation and affects cancer progression. The blockade of TGF-β signaling is excepted to realize CAFs normalization [416]. LY2157299 (galunisertib) is an oral small-molecule inhibitor of TGF-β receptor I kinase and can prevent the activation of CAFs and immunosuppression. Several trials have tested its safety and effectiveness in multiple human cancer types, both as monotherapy or in combination with other treatments, but the results have been mixed (NCT01220271, NCT01373164, NCT01246986, NCT02734160, NCT01582269) [451–458]. Another agent, minnelide, has also been studied in phased trials due to its capacity to suppress the TGF-β signaling (NCT01927965, NCT03117920).

In addition to elimination and normalization of activated CAFs, blockade of CAF-derived signalings may contributes to the acquisition of clinical benefits. FAP^+^ CAFs are identified as the primary source of a chemokine CXCL12 that exerts immunosuppressive function by binding to its receptor CXCR4 [301]. Some CXCR4 antagonists or inhibitors have been developed from bench to bed [459]. Motixafortide (BL-8040) has already been investigated in combination with pembrolizumab and/or chemotherapy in pancreatic cancer patients, showing some degree of efficacy signs (NCT02826486) [460]. AMD3100 is a CXCR4 inhibitor with the potential to reverse tumor immunosuppression and has been utilized in clinic (NCT02179970, NCT01225419). An alternative approach is to target the sonic hedgehog (SHH)-smoothened (SMO) signaling axis responsible for tumor formation and growth [461]. In several clinical trials, although a SMO inhibitor itraconazole showed antitumor activity with inhibited neoplastic growth (NCT01108094) [462], another SMO inhibitor vismodegib yielded limited or even disappointing clinical outcomes (NCT01700049, NCT01064622, NCT01195415, NCT00887159) [463–466]. LDE225 (sonidegib) is an oral small-molecule SMO inhibitor and are currently undergoing clinical assessment (NCT01579929, NCT01954355, NCT02027376) [467–469].

Overall, CAFs are increasingly recognized as an attractive target that can be clinically intervened for therapeutic benefit in cancer patients. Nevertheless, the clinical effects of targeting CAFs are not extremely encouraging and satisfactory, and no CAF-specific mAb or inhibitor has been approved for standardized cancer treatment thus for. Notably, we face numerous challenges in this field, and more in-depth investigations are still needed. First, CAFs are populations with heterogeneity and plasticity and lack definitive surface biomarkers, so it is difficult to precisely and roundly target these cells. Second, CAFs have been confirmed to possess both tumor-promoting and tumor-restraining functions depending on the TME to which they are exposed, which may account for the clinical failure of CAF-targeted therapy, or in other words, targeting CAFs in cancer is a double-edged sword and sometimes cannnot enhance tumor control as initially hoped. In this condition, developing an approach specifically targeting the tumor-promoting CAFs subtypes may be valuable. Finally, our current researches are largely at the preclinical stages, and it is clear that, to expedite the leap from bench to bedside, we still have a long way to go.

### MSCs as potential therapeutic target

TA-MSCs are widely appreciated for playing multiple roles in tumorigenesis and malignant progression, which theoretically provides new opportunities for designing feasible anticancer therapies. However, similar to CAFs populations, the lack of specific cell surface markers and controversial roles with both pro- and anti-tumorigenic functions make it challenging to target TA-MSCs precisely. Alternative strategies have been developed, among which, inhibiting MSC-related signaling pathways or secretory factors and using MSCs as a vehicle for therapeutic delivery represent promising directions [190].

The homing of TA-MSCs into the stroma can accelerate tumor growth and metastasis, and thus inhibiting TA-MSCs aggregation might potentially aid tumor control. The CXCL12/CXCR4 axis is a classic signaling that governs the homing of MSCs and upregulates the expression of PD-L1 to mediate selective immunosuppression within a tumor [470–473]. Hence, much attention has been paid to blocking this pathway with various methods. Olaptesed pegol (ola-PEG) is a high-affinity L-RNA Spiegelmer to CXCL12 with the ability to neutralize CXCL12 activity. The use of ola-PEG delays tumor growth and distant colonization of multiple myeloma cell [474]. Another approach is to target CXCR4 with specific antagonists such as AMD3100. The administration of AMD3100 not only reduced the migration potential of MSCs but also significantly enhanced the effects of anti-PD-L1 treatment [301, 475, 476].

Several previous studies determined the immunosuppressive peculiarity of TA-MSCs that exert profound influence on the growth and aggressive behavior of cancer cells, which is mainly achieved by producing immunoregulatory factors [477]. Among those secretory factors, IDO was found to be overexpressed in tumor, mediate immune escape by reducing both tumor-infiltrating CD8^+^ T cells and B cells, and contribute to the resistance of anti-CTLA-4 therapy [478–481]. Accumulated preclinical evidence is paving the way for future clinical evaluation, and currently using IDO inhibitors (e.g. navoximod and 1-methyl-DL-tryptophan) to treat cancer patients is undergoing phase clinical trials [482, 483].

An emerging therapeutic paradigm is to develop MACs as carriers for anti-tumor payloads due to their inherent tumor-homing capacity, which can also potentially attenuate their viability and invasive characteristics [484]. Genetically modified MSCs can realize the delivery of therapeutic proteins, cytokines as well as micro RNAs and has manifested obvious antitumor effects in preclinical studies. Based on the significant success of using IFNs, a class of cytokines with antitumor properties, to fight against various cancer types, engineered MSCs with the transfection IFN-α or IFN-β have been designed and exhibited varying degrees of anti-tumor activity. This strategy restricted tumor growth by inducing apoptosis and enhanced both NK cells and CD8^+^ T cells activity to reinforce antitumor immune responses [485, 486]. Other cytokines have also been stably transduced in MSCs and yielded similar antitumor outcomes, such as IL-12 and IL-17 [487, 488]. Tumor necrosis factor related apoptosis-inducing ligand (TRAIL) is a type II membrane-bound protein capable of inducing apoptosis in various cancer cells. TRAIL-expressing MSCs exhibited directional migration and infiltration toward tumor tissues, extended animal survival and contributed to overcoming drug resistance [489–491]. Furthermore, exogenous microRNAs delivered by MSCs also assist in antitumor therapy, which deserves further clinical evaluation [492–494]. Another valid use of modified MSCs is to load various anti-tumor drugs, which has been extensively tested in numerous cancer types with significant inhibiting tumor growth and improving the anti-cancer efficacy of chemotherapeutic drugs [495–497].

Although it is difficult to deplete TA­MSCs directly, the preclinical studies regarding TA-MSCs-associated factors or modified MSCs vastly motivated the initiation of a series of clinical trials (Supplementary Table 4). The clinical trials registered on *ClinicalTrials.gov.* involve inhibiting secretory factors derived from TA-MSCs, using MSCs as therapeutic agents to treat cancer patients directly and using MSCs as carrier for delivering therapeutic cytokines or proteins. Most of these studies are aimed to evaluate the safety, maximal tolerated and anti-tumor activity of MSCs loaded with different drugs in patients with several cancer types. The others tested the homing of BM-MSCs to tumor sites and the capacity of MSCs to improve the overall survival of patients (NCT01045382, NCT01983709).

In short, TA-MSCs have a multifaceted involvement in cancer, which leads to the springing up of a broader range of studies with respect to MSCs-based anticancer therapies. However, trying to realize and accelerate the clinical transformation of MSCs-based therapies remains a challenge we need to solve. Future research may focus on understanding the interplay of tumor cells and TA-MSCs for better improving clinical safety and outcomes of MSCs-based treatment (Table 4).

**Table 4:** Clinical trials based on MSCs

Target	Drug or intervation	Combination	Condition	Phase	Status	Trial number
IDO	Navoximod	No	Solid Tumor	I	Completed	NCT02048709
	Navoximod	Atezolizumab	Locally Advanced or Metastatic Solid Tumors	I	Completed	NCT02471846
Engineered MSC	BM-MSC-INFβ	No	Ovarian cancer	I	Completed	NCT02530047
	MSC-TRAIL	No	Adenocarcinoma of lung	I/II	Recruiting	NCT03298763
	GX-051	No	Head and neck cancer	I	Unknown	NCT02079324
	CELYVIR	No	Metastatic and refractory tumors	I/II	Completed	NCT01844661
	AdMSC-MV-NIS	No	Ovarian, primary peritoneal or fallopian tube cancer	I/II	Recruiting	NCT02068794
	AloCELYVIR	No	Diffuse Intrinsic Pontine Glioma	I/II	Recruiting	NCT04758533
MSC-derived exosome	iExosomes	No	Metastatic pancreas cancer	I	Recruiting	NCT03608631
Tissue-derived MSC	HB-adMSCs	No	Pancreatic cancer	I	Unknown	NCT04087889
	EB-CMF	No	Mandible tumor	I/II	Recruiting	NCT03678467
	Cord blood MSCs	NeuroRegen Scaffold™	Rectal cancer	I/II	Recruiting	NCT02648386
	MSCs	Hematopoietic stem cells	Leukemia, lymphoma, and myeloma	II	Terminated	NCT01045382
	IFNγ-primed bone marrow MSCs	No	Acute leukemia	I	Recruiting	NCT04328714

### Targeting pericytes

Apart from their acknowledged participation in maintaining the integrity of blood vessels, pericytes also act as important regulators of cancer initiation and progression. Targeting pericytes for cancer treatment is growing vigorously and exhibits some degree of benefits in preclinical studies.

One approach to target pericytes is the use of ibrutinib that not only improves the permeability of blood-brain barrier but also prolongs animal survival by enhancing chemotherapeutic effectiveness [498]. Some other investigations have confirmed that the inhibition of PDGFRβ^+^ pericytes with imatinib (a specific tyrosine kinase inhibitor) achieved pericytes depletion and delayed lymphoma growth in both murine allograft and human xenograft models [499, 500]. Clinically, the main purpose of applying imatinib is to inhibit tumor angiogenesis, but this scheme often showed modest or no effect as a single agent or in combination with chemotherapy (NCT01738139, NCT00785785) [501, 502]. In this context, dual-targeting VEGFR and PDGFR-β blockade may have better outcomes for cancer treatment and needs further clinical verification [503]. Furthermore, pericytes can protect tumor cells from immune surveillance and attack, which suggests that pericytes can potentially be viewed as a novel target or combined option for cancer immunotherapy. Designing novel agents addressing pericytes and rationally choosing therapeutic combinations are expected to improve a better cancer control and remission.

## Conclusions and perspectives

It is traditionally recognized that cancer is a malignant cell-centric disease, and with the rapid advances in the knowledge of the TME, this view is currently being replaced by the understanding of dependency and interplay between cancer cells and the tumor stroma. Specifically, cancer initiation, progression and metastasis usually elicit a broad spectrum of dynamic evolutions and alterations in host tissues, which contributes to the establishment of complicated stromal surroundings that are a prerequisite for tumor cell invasion and metastasis. These abundant stromal elements operate with each other in a coordinated fashion. Some stromal components and their molecular changes can also be considered potential biomarkers for diagnosis, prognosis, and response to treatment in cancer, which endows them with clinical significance as the abundance of stromal cells is typically related to unfavorable prognosis.

The tumor stroma participates in nearly all stages of malignant disease progression, and thereby constituting legitimate targets for therapeutic intervention. At present, a great variety of stroma-targeted modalities that aim to reduce or deprive the pro-tumorigenic functions of stroma have been developed and tested in both preclinical studies and clinical trials. Furthermore, it is possible to hopefully slow or reverse tumor development by “normalizing” the tumor stroma, such as vascular normalization and CAFs normalization. Despite these advances and findings, stromal targeting approaches can only reduce tumor growth rates or slightly extend patients survival, and are rarely curative. Simultaneously, there are still several unresolved aspects deserving further exploration. First, the stroma has both pro- and anti-tumorigenic properties since complete ablation of the stroma leads to a more invasive tumor phenotype and reduces animal survival, and thus a crucial consideration that cannot be ignored is to suitably target tumor-promoting stromal populations without hurting healthy tissues. Second, some cellular components of stroma such as CAFs and TA-MSCs lack specific cell surface markers and even possess diverse subtypes. The next goal of preclinical studies is to identify the relevant stromal cells with a specific biomarker and explore the totality tumor-promoting or tumor-restraining functions of a given stromal cell type, which will dramatically motivate the development of stroma-based therapies. Third, current mechanistic and functional investigations regarding roles of stromal elements in cancer largely rely on xenograft or syngeneic animal models, and in this condition, measures should be taken in future studies to improve the model design and try to expedite the leap from bench to bedside. Finally, in the field of cancer targeted therapies, combinational strategies with chemotherapy or immunotherapy typically exhibit more beneficial and effective outcomes. Furthermore, interfering with cancer-stromal interactions should choose optimal opportunity such as earlier phases of carcinogenesis rather than only the invasive stage, which contributes to therapeutic intervention and reduces deleterious effects. Consequently, searching for the best administration regimens with distinct combinations together with legitimate sequence and time is expected to yield significant clinical benefits for patients.

In summary, all cellular and noncellular components of the tumor stroma can interact and engage in highly regulated reciprocal dialogues, which contributes toward cancer initiation, progression and therapeutic resistance. Importantly, these findings and insights bring stroma-targeted therapies for cancer treatment onto the agenda. An in-depth understanding of the crosstalk between stroma and cancer cells is crucial for designing novel strategies for new therapeutic interventions, especially for those stroma-rich cancer types such as pancreatic carcinoma. Moving forwards, despite much work remaining to be done, it can be anticipated that, as an emerging strategy for cancer treatment, stroma-targeted therapy will open a new avenue of research in the management of malignancy and reshape the therapeutic landscape with the potential to bring more clinical benefits for cancer patients.

## Data Availability

Not applicable.

## Abbreviations

TME: Tumor microenvironment
ECM: Extracellular matrix
CAFs: Cancer-associated fibroblasts
MSCs: Mesenchymal stromal cells
TA­MSCs: Tumor­associated MSCs
TGF-β: Transforming growth factor-β
VEGF: Vascular endothelial growth factor
HGF: Hepatocyte growth factor
OSM: Oncostatin M
LOX: Lysyl oxidase
LOXL2: Lysyl oxidase like-2
RSU-1: Ras Suppressor-1
MMPs: Matrix metalloproteinases
PDAC: Pancreatic ductal adenocarcinoma
MVD: Microvessel density
HIF: Hypoxia-inducible factor
TECs: Tumor-associated endothelial cells
PGE2: Prostaglandin E2
Treg: Regulatory T cell
PD-1: Programmed death 1
PD-L1: PD-1 ligand 1
PD-L2: PD-1 ligand 2
ICC: Intrahepatic cholangiocarcinoma
OXPHOS: Oxidative phosphorylation
BC: Breast cancer
GC: Gastric cancer
IL-1β: Interleukin‑1β
TNF: Tumor necrosis factor
PMN: Pre-metastatic niche
IGF: Insulin-like growth factor
CTCs: Circulating tumor cells
cCAFs: Circulating-CAFs
PDGF: Platelet-derived growth factor
MDSCs: Myeloid-derived suppressor cells
SFRP2: Secreted frizzled-related protein 2
TAMs: Tumor‑associated macrophages
TANs: Tumor‑associated neutrophils
DCs: Dendritic cells
MCs: Mast cells
NK: Natural killer
IDO: Indoleamine 2,3-dioxygenase
CTLs: Cytotoxic T lymphocytes
PFS: Progression-free survival
OS: Overall survival
ORR: Objective response rate
CTGF: Connective tissue growth factor
ED-A: Extra domain-A
EGFR: Epidermal growth factor receptor
TKIs: Tyrosine kinase inhibitors
Ang: Angiopoietin
CAR: Chimeric antigen receptor
VDR: Vitamin D receptor
ATRA: All-transretinoic acid
PSC: Pancreatic stellate cells
